# The Mitochondrial Citrate Carrier (SLC25A1) Sustains Redox Homeostasis and Mitochondrial Metabolism Supporting Radioresistance of Cancer Cells With Tolerance to Cycling Severe Hypoxia

**DOI:** 10.3389/fonc.2018.00170

**Published:** 2018-05-25

**Authors:** Julian Hlouschek, Christine Hansel, Verena Jendrossek, Johann Matschke

**Affiliations:** Institute of Cell Biology (Cancer Research), University Hospital Essen, University of Duisburg-Essen, Essen, Germany

**Keywords:** redox homeostasis, SLC25A1, radiation resistance, chronic hypoxia, cell metabolism, mitochondria, DNA repair

## Abstract

Pronounced resistance of lung cancer cells to radiotherapy and chemotherapy is a major barrier to successful treatment. Herein, both tumor hypoxia and the upregulation of the cellular antioxidant defense systems observed during malignant progression can contribute to radioresistance. We recently found that exposure to chronic cycling severe hypoxia/reoxygenation stress results in glutamine-dependent upregulation of cellular glutathione (GSH) levels and associated radiation resistance opening novel routes for tumor cell-specific radiosensitization. Here, we explored the role of the mitochondrial citrate carrier (SLC25A1) for the improved antioxidant defense of cancer cells with tolerance to acute and chronic severe hypoxia/reoxygenation stress and the use of pharmacologic SLC25A1 inhibition for tumor cell radiosensitization. Exposure to acute or chronic cycling severe hypoxia/reoxygenation stress triggered upregulated expression of SLC25A1 in lung cancer, prostate cancer, and glioblastoma cells *in vitro*. Interestingly, exposure to ionizing radiation (IR) further promoted SLC25A1 expression. Inhibition of SLC25A1 by 1,2,3-benzene-tricarboxylic acid (BTA) disturbed cellular and mitochondrial redox homeostasis, lowered mitochondrial metabolism, and reduced metabolic flexibility of cancer cells. Even more important, combining IR with BTA was able to overcome increased radioresistance induced by adaptation to chronic cycling severe hypoxia/reoxygenation stress. This radiosensitizing effect of BTA-treated cells was linked to increased reactive oxygen species and reduced DNA repair capacity. Of note, key findings could be reproduced when using the SLC25A1-inhibitor 4-Chloro-3-[[(3-nitrophenyl)amino]sulfonyl]-benzoic acid (CNASB). Moreover, *in silico* analysis of publically available databases applying the Kaplan–Meier plotter tool (kmplot.com) revealed that overexpression of SLC25A1 was associated with reduced survival of lung cancer patients suggesting a potential link to aggressive cancers. We show that SLC25A1 can contribute to the increased antioxidant defense of cancer cells allowing them to escape the cytotoxic effects of IR. Since upregulation of SLC25A1 is induced by adverse conditions in the tumor environment, exposure to IR, or both pharmacologic inhibition of SLC25A1 might be an effective strategy for radiosensitization of cancer cells particularly in chronically hypoxic tumor fractions.

## Introduction

More than 50% of NSCLC patients receive radiotherapy (RT) or radiochemotherapy (RCT) as part of their treatment. Recent meta-analysis revealed that tumor hypoxia is major biological barrier to successful chemotherapy, RT, and potentially some targeted therapies, promoting treatment failure and poor prognosis of patients suffering from non-small cell lung cancer (NSCLC) (1). Though targeting hypoxia-mediated therapy resistance is considered as an attractive approach to improve therapy outcome in solid human tumors including NSCLC, so far clinical trials evaluating the use of hypoxia-targeting agents did not meet the expectations as they failed to reveal a benefit for the patients (1). This might at least be partially due to the lack of appropriate predictive biomarkers for patient selection but emphasizes the need for the definition of mechanism-based more effective novel therapeutic strategies to overcome hypoxia-induced therapy resistance and the co-development of predictive biomarkers and improved imaging of heterogeneous tumor hypoxia to guide RT protocols (1).

Tumors form a complex microenvironment by co-opting various normal tissue cells and immune cells to support their growth and survival (2). However, the imbalance between cell growth and tumor vascularization limits not only the availability of nutrients and oxygen (O_2_) but also the removal of secreted potentially toxic metabolites such as lactic acid. As a consequence of rapid proliferation, poor blood supply and altered metabolism tumor hypoxia is frequently linked to lactic acidosis and thus a low pH in the tumor (3, 4). The resulting O_2_-deprived and nutrient-deprived acidic microenvironment exerts a selection pressure on the tumor cells thereby directing the acquisition of genetic and epigenetic alterations that allow the cancer cells to survive and to adapt to these adverse conditions during multistep carcinogenesis thereby promoting tumor growth and even metastasis (5–8). Moreover, accumulating evidence indicates that the adverse microenvironment also impacts the therapy response at multiple levels and promotes the resistance of solid tumors to chemotherapy and RT (9, 10).

Herein limited availability of O_2_ known as “tumor hypoxia” is considered as a major environmental factor driving genomic instability, malignant progression, and resistance of solid tumors to RT and chemotherapy (9–11). The cytotoxic efficacy of RT and certain DNA-damaging anticancer drugs relies on the formation of reactive oxygen species (ROS) and thus on local availability of molecular O_2_ in the tumor tissue during treatment delivery; therefore, an acute decrease in O_2_ levels as a consequence of insufficient O_2_ supply confers direct resistance by decreasing oxidative stress and therapy-induced cell killing, the so-called “oxygen-effect” (10, 12, 13). Moreover, cancer cells dispose of multiple survival pathways that allow them to adapt to acute hypoxia and survive these adverse conditions [for a review, see Ref. (14)].

But tumor hypoxia is highly dynamic with respect to its duration (short term to long term) and schedule (transient, chronic, or intermittent), and also fluctuates regionally presumably as a result of the instability and chaotic organization of the tumor vasculature (15–18). Thus, considerable fractions of human vascularized solid tumors are exposed to dynamic changes between hypoxia and intermittent reoxygenation. Chronic changes between hypoxia and intermittent reoxygenation (“cycling hypoxia”) constitute a major driving force in the development of malignant progression, tumor heterogeneity, and clonal evolution of therapy-resistant cells (15, 19–24). Acute hypoxia/reoxygenation stress and tumor cell adaptation to chronic cycling severe hypoxia/reoxygenation stress also impact the outcome of cancer RT (23–26). A detailed understanding of the processes that allow cancer cells to escape the cytotoxic effects of RT in the adverse tumor microenvironment is required if we aim to develop effective therapeutic strategies to improve therapy outcome.

In this context, others and we demonstrated that adaptation to chronic hypoxia/reoxygenation stress drives upregulation of cellular GSH levels to avoid excessive ROS damage and promote death resistance (24, 27, 28). Altered nutrient and energy metabolism is one of the emerging hallmarks of cancer cells (29, 30). Moreover, a progressive upregulation of cellular antioxidant systems has been associated with malignant progression (31), suggesting a broader relevance of the above findings for understanding tumor progression and clonal evolution of therapy-resistant cells in tumors with heterogeneous environments. Importantly, as a proof of principle others and we demonstrated that targeting metabolic reprogramming associated with increased GSH levels is a promising strategy for radiosensitization (24, 32).

However, the above studies also demonstrated that cancer cells use different metabolic adaptation strategies to increase their cellular GSH levels, avoid ROS-dependent damage, and escape genotoxic therapies, presumably depending on the genetic background (24, 28, 31–33). Thus, targeting altered glutamine usage will be a viable therapeutic strategy to reduce glutathione levels in some but not all cancer models (24). We therefore reasoned that targeting the increased GSH-based antioxidant capacity and thus the common phenotype of aggressive cancer cells with increased stress tolerance, might be an effective strategy for therapeutic intervention with broader relevance.

Generally, increased GSH levels can be targeted by using drugs interfering with the regeneration of glutathione, the provision of reduction equivalents, increased glutathione synthesis, or glutathione transport and uptake (24, 34, 35). In an effort to define novel ways to specifically target increased GSH levels in aggressive cancer cells the observation about a link between the mitochondrial citrate carrier (CIC, also known as mitochondrial citrate transport protein, CTP) and the maintenance of cytosolic and mitochondrial NADPH pools and the mitochondrial redox homeostasis attracted our attention (36, 37).

The CIC is encoded by the *SLC25A1* gene located on chromosome 22q11.2. Besides citrate, SLC25A1 is also responsible for the electroneutral transport of isocitrate, malate, and phosphoenolpyruvate (38). Furthermore, SLC25A1—together with cytosolic isocitrate dehydrogenase 1 (IDH1) and mitochondrial isocitrate dehydrogenase 2 (IDH2)—takes part in the transport of NADPH derived from reductive carboxylation over the mitochondrial membrane (36) and might thus play a role in GSH regeneration. Overall, SLC25A1 is important for the maintenance of mitochondrial homeostasis and its overexpression was shown to drive tumorigenesis in various types of cancer (39).

Though the authors linked SLC25A1 expression to anchorage-independent growth of NCI-H460 cancer cells (36), it was tempting to speculate that the function of SLC25A1 regarding maintenance of redox homeostasis and mitochondrial function might contribute to the increased radioresistance of lung cancer cells with tolerance to chronic hypoxia/reoxygenation stress. However, the role of SLC25A1 for the cellular radiation response has not yet been investigated. Therefore, in the present study we aimed to explore the role of SLC25A1 for the increased antioxidant capacity of cancer cells adapted to chronic cycling severe hypoxia/reoxygenation stress and the use of SLC25A1 inhibition as novel strategy for radiosensitization of NCI-H460 lung adenocarcinoma cells exposed to acute or chronic cycling severe hypoxia.

## Results

### Acute and Chronic-Cycling Hypoxia Increase Expression of *SLC25A1* and *IDH2*

**Figure 1:**
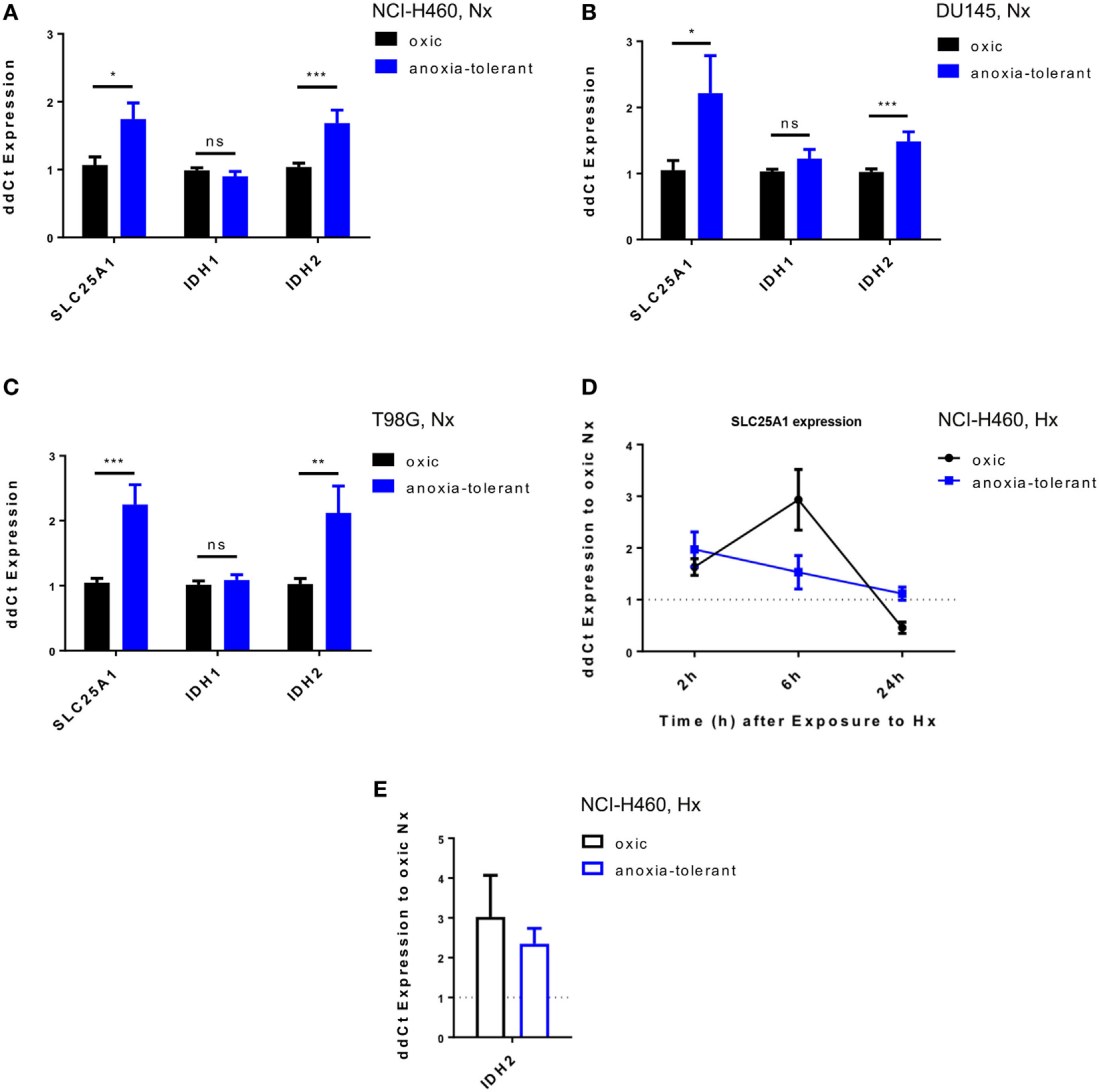
Exposure of cancer cells to acute or chronic cycling severe hypoxia leads to upregulated expression of *SLC25A1* and *IDH2*. Expression of *SLC25A1*, isocitrate dehydrogenase 1 (*IDH1*; cytosolic), and isocitrate dehydrogenase 2 (*IDH2*; mitochondrial) was determined in anoxia-tolerant cancer cells generated by exposure to 16 cycles (T98G) or 25 cycles (NCI-H460, DU145) of severe hypoxia (48 h, <0.1% O_2_) and reoxygenation (120 h air plus 5% CO_2_ referred as 20% O_2_) and the respective oxic control cells (24) by quantitative real-time PCR analysis. Data show basal expression of *SLC25A1, IDH1*, and *IDH2* in **(A)** NCI-H460, **(B)** DU145, and **(C)** T98G oxic and anoxia-tolerant cells under normoxic (Nx) conditions (20% O_2_). Changes in *SLC25A1* [2–24 h, **(D)**] and *IDH2* [24 h, **(E)**] expression in oxic and anoxia-tolerant NCI-H460 cells in response to acute severe hypoxia (Hx, 0.2% O_2_). Data were always normalized to the respective oxic control cells under basal Nx conditions. Mean values ± SEM are shown, *n* = 3 (*p ≤ 0.05, **p < 0.01, and ***p ≤ 0.001; *t*-test).

To gain insight into a potential relevance of SLC25A1 for tolerance of lung cancer cells to chronic cycling severe hypoxia, we used our established cell model of so-called “anoxia-tolerant” NCI-H460 lung cancer cells and the respective control cells termed “oxic” NCI-H460 cells. These cells had been exposed to 25 cycles of severe hypoxia (48 h) and reoxygenation stress (120 h) as described earlier (24). Quantitative real-time PCR (qRT-PCR) analysis revealed a significant upregulation of *SLC25A1* in the anoxia-tolerant NCI-H460 cells as compared with the oxic control cells under standard culturing conditions, suggesting that basal upregulation of *SLC25A1* might be a consequence of adaptation to chronic cycling severe hypoxia (Figure 1A). *SLC25A1* upregulation was associated with upregulation of *IDH2*, whereas *IDH1* expression was not altered (Figure 1A). To test a more general relevance of these findings, we additionally examined the expression of the respective genes in similarly generated anoxia-tolerant DU145 and T98G cells and again observed an upregulated basal *SLC25A1* and *IDH2* expression in the anoxia-tolerant cells as compared to the respective oxic control cells (Figures 1B,C), whereas *IDH1* expression was not altered. Interestingly, exposure to acute severe hypoxia (0.2% O_2_) was also able to trigger increased expression of *SLC25A1* and *IDH2* in the lung cancer cells and this effect was observed in both, oxic and anoxia-tolerant NCI-H460 cancer cells, compared to the oxic NCI-H460 control cells under normoxic (Nx) conditions (Figures 1D,E). However, the apparent upregulation of *SLC25A1* and *IDH2* expression induced by acute hypoxia was not significant for anoxia-tolerant NCI-H460 cells as the major increase over the levels of oxic NCI-H460 control cells in normoxia was already caused by the adaptation to chronic cycling severe hypoxia, whereas exposure to acute hypoxia had only a minor addition effect (Figures S1D,E in Supplementary Material). Similar observations about a significant upregulation of SLC25A1 expression upon exposure of NCI-H460 cells to acute or chronic cycling severe hypoxia were made using Western blot analysis (Figures S1A,B in Supplementary Material).

### Overexpression of SLC25A1 in Lung Cancer Is Associated With Reduced Overall Survival of Lung Cancer Patients

**Figure 2:**
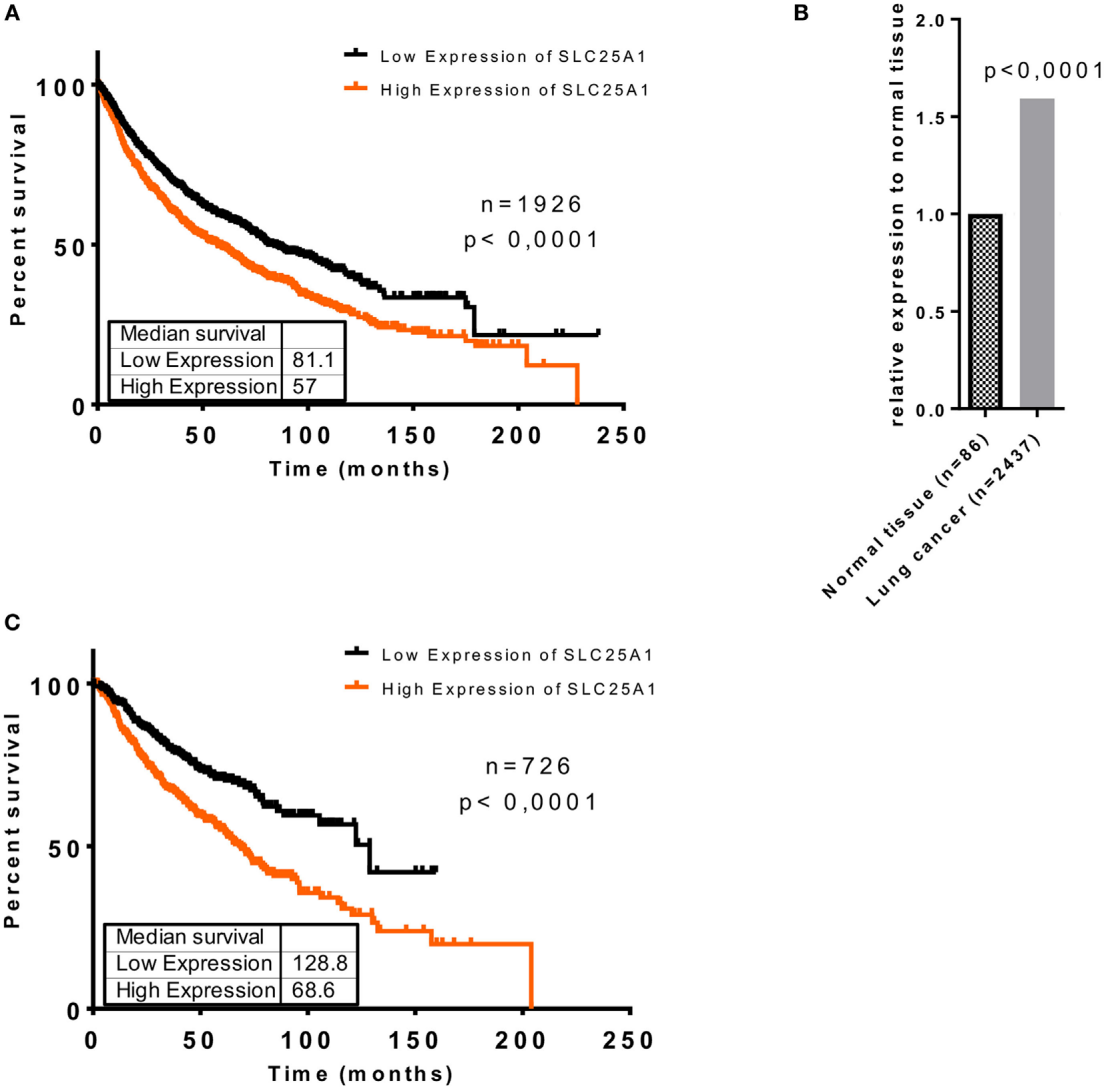
Elevated expression of SLC25A1 is associated with worse clinical outcome of lung cancer patients, particularly upon tumor resection with negative margins (R0). Clinical relevance of the SLC25A1 expression was obtained by searching and analyzing available patient array data by the Kaplan–Meier plotter tool (kmplot.com). Data show **(A)** overall survival in lung cancer patients, **(B)** relative expression of SLC25A1 in normal tissue and lung cancer, and **(C)** overall survival in lung cancer patients upon surgery with negative tumor margins (R0). For the analysis, the cohort was split by median of SLC25A1 expression (“High” and “Low,” respectively). Median survival (in months) is indicated for each survival curve.

To investigate whether upregulation of SLC25A1 in an adverse microenvironment *in vitro* might be relevant for the clinical situation, we searched for and analyzed the data of Kaplan–Meier plotter tool (kmplot.com) (40–42), about SLC25A1 expression in lung cancer patients (Figures 2A,C) and normal lung tissue by an *in silico* analysis, respectively (Figure 2B). The patient cohort has been described in detail in Ref. (40), whereas the parameters used for our *in silico* analysis are given in Tables S1 and S2 in Supplementary Material. We split the lung cancer patient cohort by median of SLC25A1 expression into “High” and “Low,” respectively. Our *in silico* analysis revealed that SLC25A1 overexpression was associated with significantly reduced overall survival and median survival in lung cancer patients. Interestingly, this effect regarding overall and median survival was enhanced in the cohort of patients with successful surgery with tumor-free margins (R0-resection) (Figure 2C). Of note, SLC25A1 displayed a higher expression in lung cancer patients compared to normal lung tissue (Figure 2B) suggesting that SLC25A1 might be a relevant target in lung cancer.

### Pharmacologic Inhibition of SLC25A1 Sensitizes Cancer Cells to the Cytotoxic Action of Ionizing Radiation (IR) and Overcomes Increased Radioresistance Induced by Chronic Cycling Severe Hypoxia

**Figure 3:**
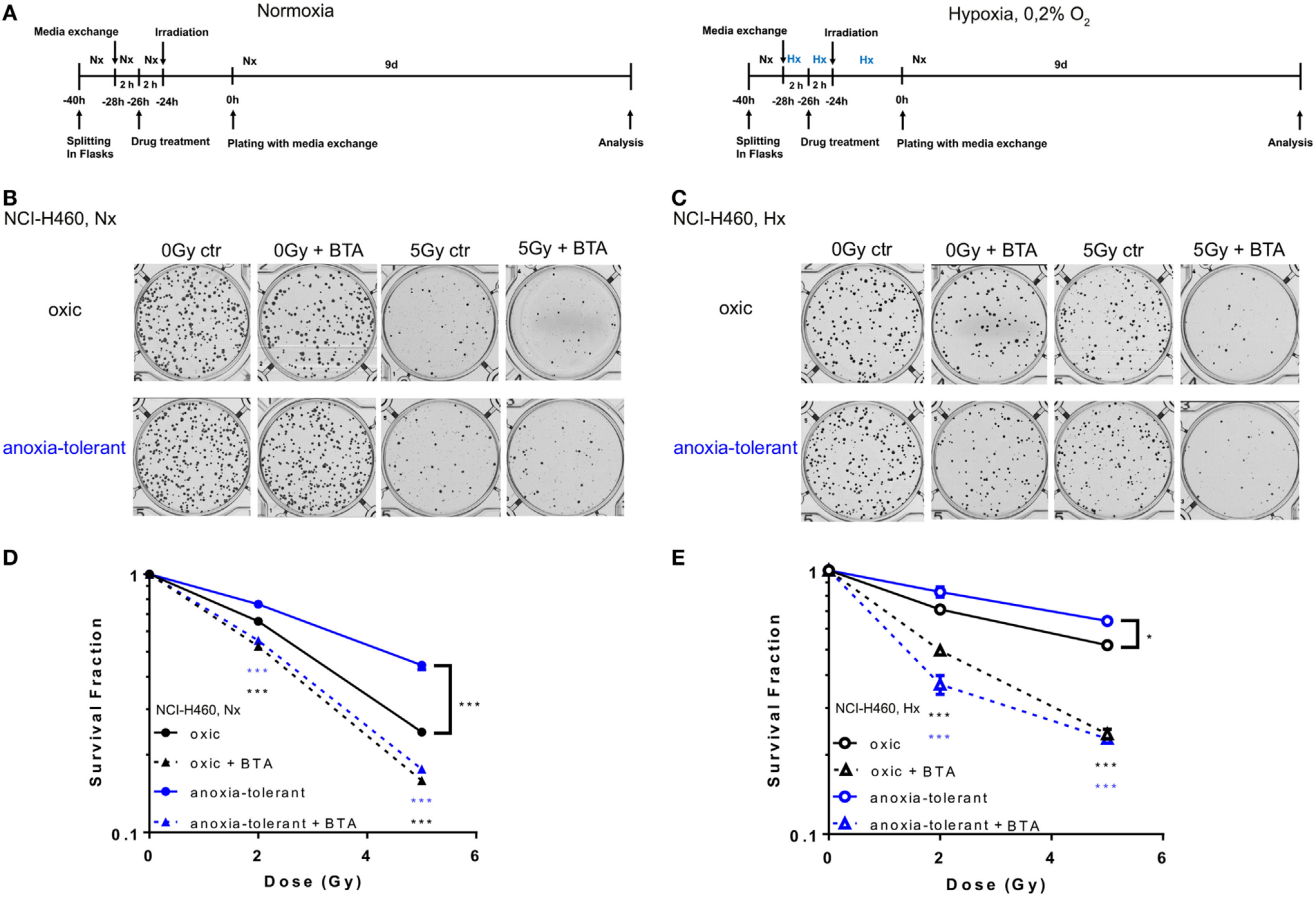
Pharmacologic inhibition of SLC25A1 overcomes chronic cycling hypoxia-induced radioresistance. Anoxia-tolerant NCI-H460 cells and the oxic NCI-H460 control cells were pretreated with 5-mM BTA (2 h prior to IR) under normoxic (Nx) conditions (20% O_2_) or upon 2-h preincubation in severe hypoxia (Hx, 0.2% O_2_). Twenty-four hours after treatment cells were collected, plated at different cell numbers (200–3,200) in full medium without the inhibitor and grown under Nx for 9 days. **(A)** Schematic representation of the experimental timeline in normoxia (Nx, left panel) or severe Hx (right panel). Photomicrographs in **(B)** and **(C)** show representative pictures of colony formation upon treatment with IR without or with prior BTA treatment for Nx **(B)** or severely hypoxic conditions **(C)**. Survival curves shown in **(D,E)** depict quantification of colony formation upon treatment in normoxia **(D)** and severe hypoxia **(E)**. Colonies were scanned and counted using GelCount. Survival fractions were calculated to the plating efficiency of unirradiated cells under Nx or Hx conditions. Mean values ± SEM are shown, *n* = 3 (*p ≤ 0.05, **p < 0.01, and ***p ≤ 0.001; two-way ANOVA with Tukey post-test.) Comparisons between treatment and corresponding controls at the same dose are indicated.

So far, our data demonstrated that acute and chronic cycling severe hypoxia alter the expression levels of SLC25A1. Moreover, our earlier data revealed that anoxia-tolerant NCI-H460 cells are more resistant to IR and chemotherapeutic agents compared to non-selected oxic control cells and this increased radioresistance could be linked to improved antioxidant defense of anoxia-tolerant cancer cells (22–24). Since SLC25A1 has recently been linked to cellular redox homeostasis (36) we wondered whether SLC25A1 might contribute to increased radioresistance of cancer cells in acute or chronic cycling severe hypoxia. To test a potential influence of SLC25A1 on radiosensitivity, we treated the anoxia-tolerant NCI-H460 cells and the oxic control cells with the pharmacological SLC25A1-inhibitor 1,2,3-benzene-tricarboxylic acid (BTA) (38, 39) 2 h before irradiation with 0–5 Gy under standard Nx conditions (20% O_2_) and determined the effects of single and combined treatment on cell survival in standard long-term colony survival assays upon removal of BTA 24 h after treatment (delayed plating) (experimental timeline, Figure 3A). To test the impact of drug-treatment in acute hypoxia, we additionally performed similar experiments in cells that had been adapted to acute severe hypoxia (0.2% O_2_) for 2 h prior to inhibitor treatment (Figure 3A).

Our results revealed that treatment with the SLC25A1-inhibitor BTA sensitized both oxic and anoxia-tolerant NCI-H460 cancer cells to the cytotoxic action of IR when treatment was performed under Nx conditions (Figures 3B,D). As expected, the effect was more pronounced in the anoxia-tolerant cells with increased SLC25A1 expression and higher radioresistance. Interestingly, radiosensitization was also observed when treatment was performed in acute severe hypoxia (Figures 3C,E). BTA treatment was even able to partially compensate the reduced efficacy of IR in acute hypoxia (Figure 3E). Remarkably, we observed these effects even though BTA was already removed 24 h after IR so that long-term incubation was performed in inhibitor-free media. To confirm the relevance of SLC25A1 inhibition for the radiorsensitizing effects of BTA, we performed additional experiments with a chemically distinct SLC25A1-inhibitor 4-Chloro-3-[[(3-nitrophenyl)amino]sulfonyl]-benzoic acid (CNASB), with higher specificity for SLC25A1 (43). These experiments revealed that combined treatment of CNASB and IR similarly sensitized both, oxic and anoxia-tolerant NCI-H460 cancer cells, to the cytotoxic action of IR under Nx and hypoxic (Hx) conditions in short-term proliferation (Figure S4A in Supplementary Material) and long-term survival assays (Figure S4C in Supplementary Material) corroborating the radisensitizing action of BTA at the level of SLC25A1.

### Cellular and Mitochondrial Redox Homeostasis Is Impaired After Inhibition of SLC25A1

**Figure 4:**
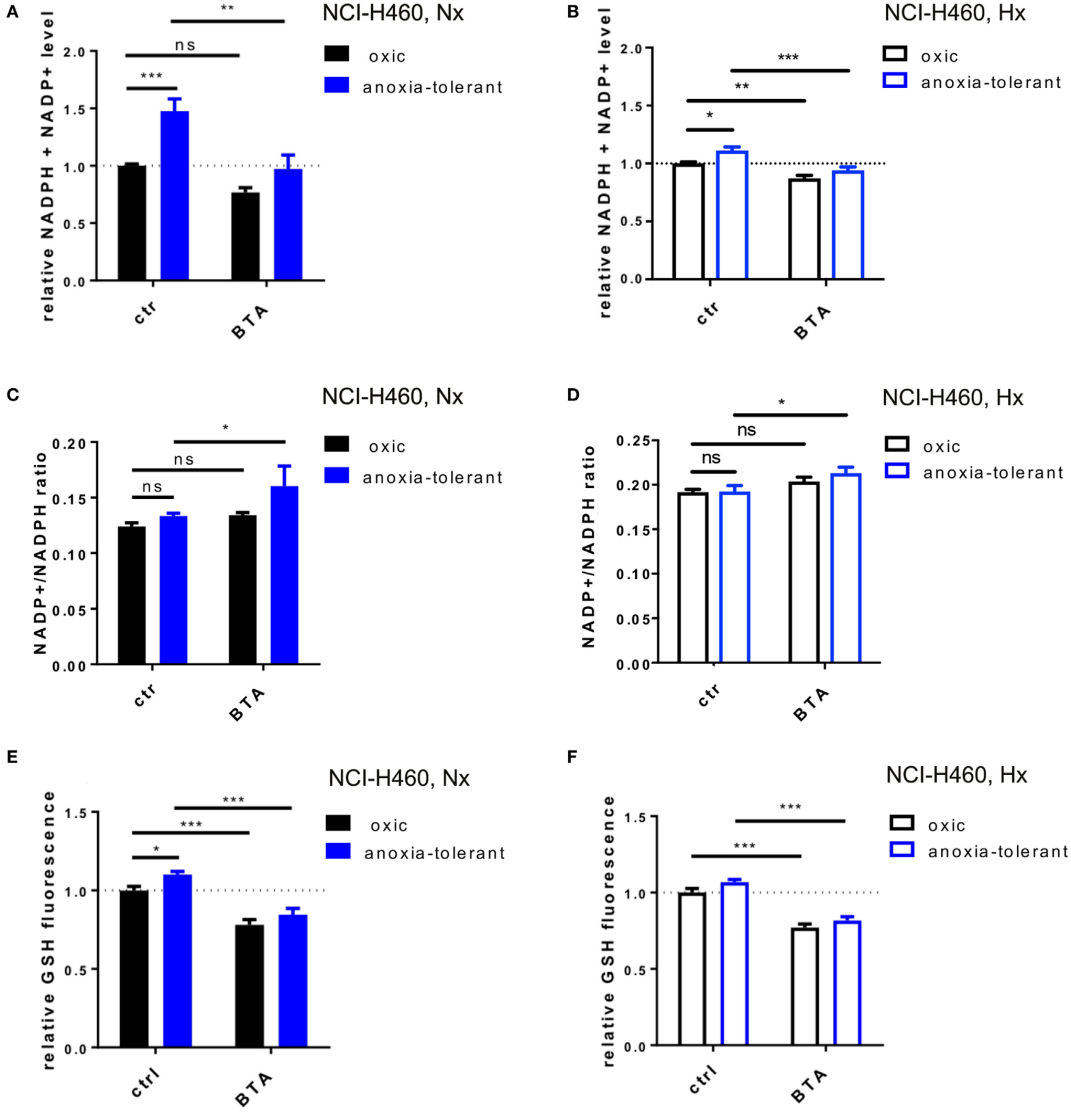

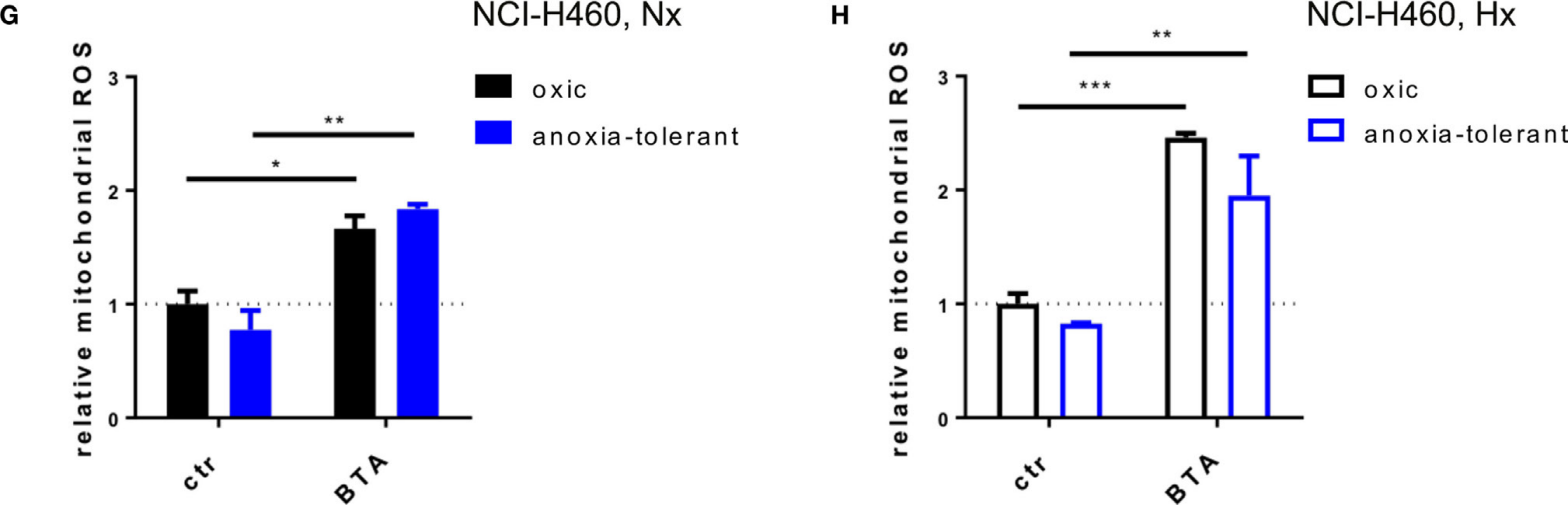
Inhibition of SLC25A1 disturbs cellular and mitochondrial redox homeostasis. NCI-H460 oxic and anoxia-tolerant cells were treated with 5 mM BTA in normoxia (Nx, 20% O_2_) or upon preincubation for 2 h in acute severe hypoxia (Hx, 0.2% O_2_) and antioxidant capacity and ROS formation were determined 2 or 6 h after treatment in functional assays. **(A)** Impact of BTA treatment on total NADPH levels (NADPH + NADP^+^) under normoxic (Nx) conditions as determined by using a luciferase-coupled enzymatic reaction 2 h after treatment. **(B)** Impact of BTA treatment on total NADPH levels (NADPH + NADP^+^) under Hx conditions. **(C)** NADP^+^/NADPH ratio upon 2 h of BTA treatment under Nx conditions. **(D)** NADP^+^/NADPH ratio upon 2 h of BTA treatment under Hx conditions. **(E)** Influence of BTA treatment on GSH levels under Nx conditions as measured by fluorescence of monochlorobimane (MCB) 2 h after treatment. **(F)** Influence of BTA treatment on GSH levels under Hx conditions. **(G)** Mitochondrial superoxide reactive oxygen species (ROS) as determined in flow cytometry by staining with MitoSox 6 h after treatment under Nx conditions. Fold changes of gated positive cells are indicated. **(H)** Mitochondrial ROS as determined in flow cytometry by staining with MitoSox 6 h after treatment under Hx conditions. Mean values ± SEM are shown, *n* = 3. (ns *p* > 0.05, *p ≤ 0.05, **p < 0.01, and ***p ≤ 0.001; two-way ANOVA with Tukey post-test.)

As demonstrated in NCI-H460 cells IDH1, IDH2, and SCL25A1 are all part of a pathway responsible for the bidirectional transport of NADPH over the mitochondrial inner membrane (36). We therefore investigated whether the cytotoxic and radiosensitizing effects of the acute BTA treatment might be linked to alteration of cellular redox homeostasis. To this end, we measured total NADPH level (NADPH + NADP^+^) by using a luciferase-coupled enzymatic reaction. As shown in Figure 4A, anoxia-tolerant NCI-H460 cells were characterized by a significant increase of total NADPH, presumably as a consequence of adaptation to chronic cycling severe hypoxia, whereas treatment with BTA for 2 h reduced the total NADPH level to the levels of the untreated oxic control cells (Figure 4A). In contrast, BTA had no significant effect on total NADPH levels in the oxic control cells (Figure 4A). We observed less pronounced effects regarding the BTA-mediated depletion of total NADPH levels in acute hypoxia (Figure 4B). Interestingly, the NADP^+^/NADPH ratios were slightly increased after BTA treatment especially in anoxia-tolerant NCI-H460 cancer cells compared to respective controls in normoxia and hypoxia (Figures 4C,D).

NADPH is required amongst others for the regeneration of the reduced form of glutathione (GSH). We therefore next examined the effect of BTA treatment on cellular glutathione levels (Figures 4E,F). In line with our previous findings (24), anoxia-tolerant NCI-H460 cancer cells had elevated GSH levels compared to oxic control cells (Figure 4E). As expected, BTA treatment significantly reduced GSH levels in oxic and anoxia-tolerant NCI-H460 cancer cells when BTA treatment was performed under Nx conditions (Figure 4E). Similar observations were made when cells were treated with BTA in acute severe hypoxia (Figure 4F). Of note, BTA decreased the GSH levels of the anoxia-tolerant cancer cells to the levels of the oxic control cells (Figures 4E,F).

To further specify the impact of the SLC25A1-inhibitor BTA on cellular antioxidant capacity, particularly mitochondrial and cellular redox homeostasis, we measured mitochondrial and cellular ROS after BTA treatment in normoxia and acute severe hypoxia (Figures 4G,H; Figure S2 in Supplementary Material). Importantly, BTA treatment increased the levels of mitochondrial ROS in oxic and anoxia-tolerant NCI-H460 cancer cells under Nx conditions (Figure 4G). This effect was even enhanced when BTA treatment was performed in acute hypoxia (Figure 4H; Figure S2 in Supplementary Material). Additionally, cellular ROS-levels were increased in both, oxic and anoxia-tolerant cells, upon BTA treatment in normoxia and acute severe hypoxia (Figures S2E,F in Supplementary Material). Nevertheless, BTA-induced cellular ROS levels were lower compared to the mitochondrial ROS levels (Figures S2 in Supplementary Material).

Taken together, the SLC25A1-inhibitor BTA efficiently decreased cellular NADPH and GSH levels resulting in increased mitochondrial and cellular ROS. These findings point to a role of SLC25A1 in the regulation of cellular antioxidant capacity and mitochondrial redox homeostasis.

### SLC25A1 Inhibition Lowers Mitochondrial Metabolism and Alters Metabolic Demand

**Figure 5:**
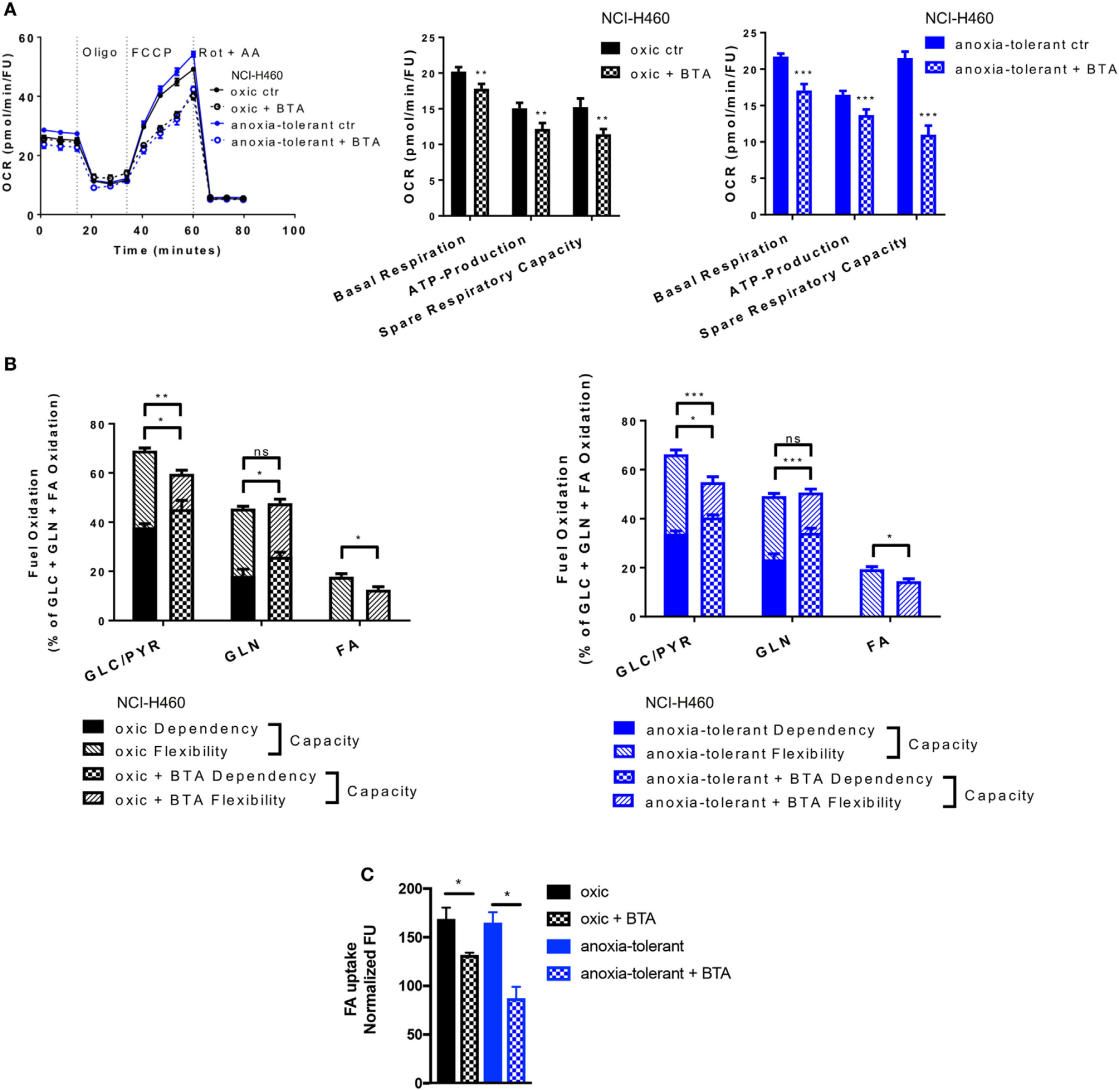
Metabolic alterations upon SLC25A1 Inhibition in NCI-H460 oxic and anoxia-tolerant cells. NCI-H460 oxic and anoxia-tolerant cells were treated for 24 h with 5 mM 1,2,3-benzene-tricarboxylic acid (BTA) under standard ambient conditions and various parameters of cell metabolism were determined by using a Seahorse XFe 96 analyzer. Fatty-acid (FA) uptake was measured by using the fluorescent FA analog C1-BODIPY^®^ 500/510 C12. **(A)** Oxygen consumption rate (OCR) was measured using Mito Stress Test Kit and normalized to Hoechst 33342 fluorescence units (FU) 24 h after treatment with BTA. Real-time Injection of oligomycin (Oligo, 1 µM), carbonyl cyanide-*4*-(trifluoromethoxy)phenylhydrazone (FCCP, 2 µM), Rotenone (Rot, 0.5 µM) and antimycin A (AA, 0.5 µM). Calculated parameters of the assay are indicated in bar graphs. **(B)** Percentage of total mitochondrial respiration for each fuel pathway measured with Seahorse XFe 96 analyzer using Fuel Flex Test Kit 24 h after treatment with BTA (fuel pathways: GLC/PYR: Glucose/Pyruvate, inhibition by UK5099; GLN, glutamine, inhibition by BPTES, FA: fatty acids, inhibition by etomoxir). **(C)** FA uptake measured after 24 h of BTA treatment. Mean values ± SEM are shown. Data in **(A)** and **(B)** from *n* = 12–18 wells from two independent experiments; Data in **(C)** *n* = 3 (*p ≤ 0.05, **p < 0.01, and ***p ≤ 0.001. **(A)** t-test; **(B)** and **(C)** two-way ANOVA with Tukey post-test).

So far, our data indicated that increased production of mitochondrial ROS upon SLC25A1 inhibition by BTA treatment might play a role in BTA-mediated radiosensitization. However, SLC25A1 is of broader relevance for the transport of metabolites between mitochondrial intermembrane space and mitochondrial matrix as it shuttles isocitrate, malate and phosphoenolpyruvate, in addition to citrate (38, 44). We therefore wondered whether BTA might have a more general effect on mitochondrial metabolism and therefore additionally measured the effects of BTA on mitochondrial metabolism by using extracellular flux measurements (Figure 5).

Pretreatment of cells with BTA for 24 h led to decreased basal mitochondrial respiration and lowered ATP-Production in both, oxic and anoxia-tolerant NCI-H460 cells, as determined by using the Mito Stress Test (Figure 5A). Additionally, we observed a decreased ability of cells treated with the SLC25A1 inhibitor to respond to forced mitochondrial respiration by uncoupling the electronic transport chain, an effect that is termed reduced spare respiratory capacity (Figure 5A, left panel). As shown in Figure 5A, the inhibitory effect was more pronounced in the anoxia-tolerant NCI-H460 cells as these cells were found to dispose of an increased spare respiratory capacity (Figure 5A, right panel) in comparison to oxic control cells (Figure 5A, middle panel). Similar effects on cell metabolism were observed when using CNASB, another more potent small molecule inhibitor of SLC25A1 (Figure S4B in Supplementary Material).

But inhibition of SLC25A1 might also impact the metabolic demands of oxic and anoxia-tolerant NCI-H460 cells to maintain mitochondrial function. To identify the metabolic pathways needed to maintain mitochondrial respiration under BTA treatment we performed the Fuel Flex Test. In this test the glucose/pyruvate pathway was inhibited by UK5099, the glutamine pathway by using BPTES and the fatty acid (FA) pathway by etomoxir treatment, respectively. Overall, BTA treatment induced a higher dependency of oxic and anoxia-tolerant NCI-H460 cells on the final glycolytic product pyruvate as well as glutamine to maintain mitochondrial respiration (Figure 5B). Furthermore, combined inhibition of two of these pathways reduced the capacity to maintain mitochondrial respiration only for glucose/pyruvate and FAs but not for glutamine when the other two pathways were inhibited (Figure 5B). This suggests that BTA reduces the capacity and flexibility of oxic and anoxia-tolerant cells to oxidize other fuels, when the pathway of interest is inhibited. Since BTA treatment lowered the flexibility of oxic and anoxia-tolerant cell to oxidize FAs, when glucose/pyruvate or glutamine pathways were inhibited, we measured FA uptake upon BTA treatment. We observed a reduction in the uptake of labeled FAs following BTA treatment (Figure 5C). Taken together, BTA treatment lowered the metabolic flexibility for all metabolic pathways examined in both oxic and anoxia-tolerant NCI-H460 cells, as defined by the difference between metabolic dependency and capacity (Figure 5B) and lead to reduced FA uptake (Figure 5C).

These findings revealed a role of SLC25A1 in conserving mitochondrial metabolism. Importantly, the enhanced flexibility of mitochondrial metabolism as a consequence of upregulated SLC25A1 expression might contribute to increased stress-tolerance of the NCI-H460 cancer cells.

### Inhibition of SLC25A1 Affects the Repair of IR-Induced Double-Strand Breaks (DSB)

**Figure 6:**
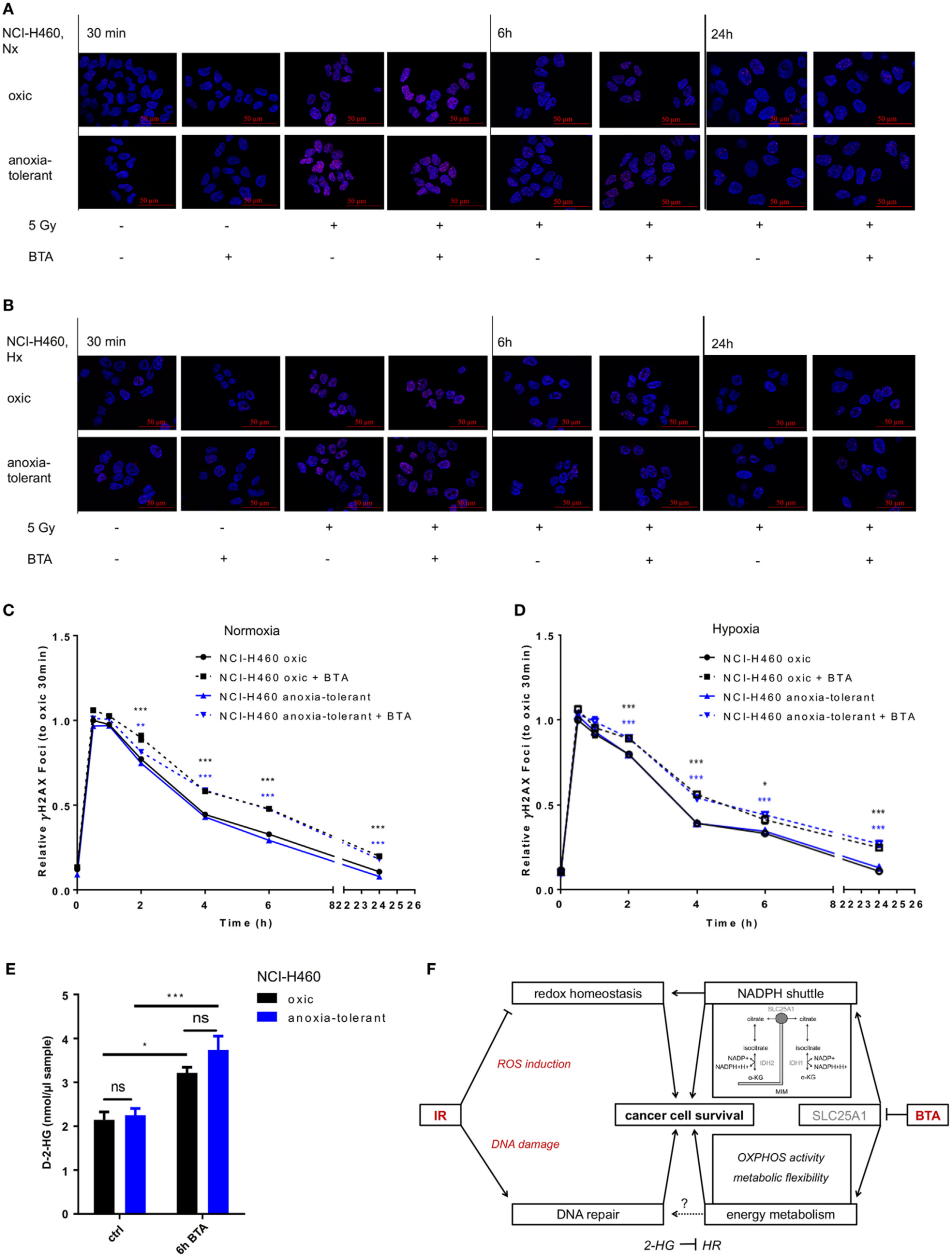
Inhibition of SLC25A1 affects the repair of radiation-induced DNA damage. NCI-H460 oxic and anoxia-tolerant cells were left untreated or exposed to ionizing radiation (IR) with a single dose of 5 Gy with or without prior preincubation of the cells for 2 h with 5 mM 1,2,3-benzene-tricarboxylic acid (BTA) under normoxic (Nx, 20% O_2_) and hypoxic (Hx, 0,2% O_2_) conditions. **(A)** Time-dependent accumulation of γH2AX foci in irradiated NCI-H460 oxic and anoxia-tolerant cells under Nx conditions was evaluated by fluorescence microscopy without and with additional treatment with 5 mM BTA at indicated time points after IR. **(B)** Time-dependent accumulation of γH2AX foci in irradiated NCI-H460 oxic and anoxia-tolerant cells under Hx conditions was evaluated by fluorescence microscopy without and with additional treatment with 5-mM BTA at indicated time points after IR. **(C)** Mean number of γH2AX foci per cell after IR without and with additional BTA treatment in Nx was calculated with the Focinator v2 software and normalized to oxic control cells 30 min after IR. **(D)** Mean number of γH2AX foci per cell after IR without and with additional BTA treatment in Hx was calculated with the Focinator v2 software and normalized to oxic control cells 30 min after IR. **(E)** D-2-hydroxyglutarate levels (D-2-HG) were determined in lysed cells 6 h after treatment with BTA in Nx. **(F)** Schematic representation of the suggested mechanisms of the actions of BTA and IR on cancer cell survival. Mean values ± SEM are shown, *n* = 3 (*p ≤ 0.05, **p < 0.01, and ***p ≤ 0.001; two-way ANOVA with Tukey post-test. Comparisons between treatment and corresponding controls at the same time point are indicated). MIM, mitochondrial inner membrane; α-KG, α-Ketoglutarate; HR, homologous recombination.

So far, our data indicated that pharmacologic inhibition of SLC25A1 by BTA or CNASB increases radiosensitivity of oxic and anoxia-tolerant NCI-H460 cancer cells and that disturbance of the redox homeostasis and of metabolic flexibility might participate in the radiosensitizing effects. Herein, one important aspect of radiosensitivity is the ability of the cells to repair IR-induced DNA DSB. Therefore, we finally examined if BTA treatment would affect the time-dependent induction and resolution of DNA-repair foci positive for Histone H2A.X phosphorylated at serine 139 (γH2AX), a marker for DNA DSB (45). BTA treatment alone (without IR) did not induce additional DSB, ruling out a direct effect of BTA on DNA-damage induction and repair (Figures 6A,B). Interestingly, the presence of BTA during exposure to IR slowed the resolution of IR-induced γH2AX-foci and led to higher residual amount of DNA lesions 24 h after IR-treatment in Nx (Figures 6A,C) and even in Hx conditions (Figures 6B,D). Surprisingly, despite the differences in SLC25A1 expression between oxic and anoxia-tolerant NCI-H460 cells, BTA treatment had similar effects on DSB repair in both cell lines. Even more important, despite the obvious differences in radiosensitivity, the kinetics of DSB repair as determined by the resolution of IR-induced γH2AX-foci were rather similar in oxic and anoxia-tolerant NCI-H460 cells. This suggests that other parameters of the DNA damage response might dictate the differences in radiosensitivity, presumably the differences in the ability to cope with radiation-induced ROS. Nevertheless, it was an interesting observation that BTA was able to retard the resolution of IR-induced γH2AX-foci and cause a higher amount of residual DNA lesions 24 h after irradiation.

Interestingly, it had recently been shown that a knockout of SLC25A1 not only impairs mitochondrial function, resulting in higher glycolysis rate and the usage of glutamine for compensation of impaired FA synthesis, but also leads to the accumulation of 2-hyodroxyglutarate (2-HG) in NCI-H460 cells (44). Of note, accumulation of the oncometabolite 2-HG has been linked to DNA-repair defects (46). We therefore wondered whether BTA-mediated inhibition of SLC25A1 might have similar effects and measured the effects of BTA treatment on the level of D-2-HG. For this, we used an enzymatic reaction detecting a colored product photometrically at 450 nm. As shown in Figure 6E, BTA treatment indeed significantly increased the levels of D-2-Hydroxyglutarate (D-2-HG) 6 h after BTA treatment in both oxic and anoxia-tolerant NCI-H460 cells (Figure 6E). This increase in 2-HG might thus be responsible for the observed delay in the kinetics of the repair of radiation-induced DNA DSB observed in both cell lines (Figures 6A–D). The finding that there was no significant difference in the absolute D-2-HG levels upon BTA treatment between oxic control and anoxia-tolerant NCI-H460 might explain why the BTA-induced retardation in DNA repair was similar in both cell lines. Taken together our novel findings suggest that BTA exerts a dual effect on the cellular radiation response by targeting both DNA repair and metabolic escape mechanisms of aggressive cancers making it a promising radiosensitizer.

## Discussion

Tumor hypoxia is an important biological factor causing poor therapy outcome and worse prognosis in NSCLC patients. However, so far hypoxia-targeting strategies have not been translated into clinical practice highlighting the need for an improved understanding of the underlying mechanisms if we aim to develop more effective strategies for therapeutic intervention. In our work, we focus on mechanisms of radiation resistance caused by adaptation of cancer to an adverse Hx environment.

Our previous work identified enhanced antioxidant capacity based on glutamine-dependent glutathione-regeneration as a mechanism favoring stress tolerance and radioresistance of cancer cells with tolerance to chronic cycling severe hypoxia (23, 24). Here, we reveal important novel mechanistic aspects of increased antioxidant capacity in cancer cells and demonstrate a therapeutic potential for SLC25A1 inhibition to overcome radioresistance in chronically Hx lung cancer cells: (i) Exposure of cancer cells to acute or chronic cycling severe hypoxia was associated with upregulated expression of the citrate transporter SLC25A1. (ii) Pharmacologic inhibition of SLC25A1 by BTA reduced cellular antioxidant capacity, enhanced the generation of mitochondrial ROS and abrogated the increase in radioresistance of lung cancer cells induced by adaptation to chronic cycling severe hypoxia. (iii) BTA treatment was also associated with a pronounced disturbance of mitochondrial metabolism, accumulation of the oncometabolite 2-HG, and impaired repair of radiation-induced DNA DSB. These findings point to a yet unknown role of SLC25A1 in radioresistance that might be linked to regeneration of GSH for ROS-detoxification and metabolic regulation of DNA repair. Remarkably, we confirmed the metabolic and radiosensitizing effects of SLC25A1-inhibition also with CNASB, another potent small-molecule inhibitor with documented higher specificity to SLC25A1 (43). In line with our observations using BTA the radiosensitizing effects of CNASB were more prominent in the anoxia-tolerant cancer cells. This further corroborates our assumption that chronic cycling hypoxia/reoxygenation stress increases not only the expression but also the reliance of cancer cells on the SLC25A1 mediated redox-homeostasis for survival under stress conditions.

Interestingly, high SLC25A1 expression in lung cancer patients has already been linked with poorer overall survival compared to lung cancer patients with low SLC25A1 expression in an earlier report (42). However, here we expand these findings with respect to the importance of SLC25A1 in terms of recurrence and progression of lung cancer suggesting that SLC25A1 may be an attractive therapeutic target for tumor cell-specific radiosensitization at least in patients with high SLC25A1 expression. Of note, mutant p53 can promote transcriptional activation of SLC25A1 eventually by an interaction with the FOXO-1 transcription factor (42). This suggests that SLC25A1 might mediate some of the oncogenic activities of mutant p53—an important prognostic marker predictive for relapse and resistance to chemotherapy and RT (47).

In addition, upregulated expression of SLC25A1 was always associated with upregulation of IDH2. Thus, SLC25A1 and IDH2 might cooperate in adaptive metabolic processes that allow cancer cells to cope with the adverse conditions in the tumor microenvironment. Others have described a crucial role of the presence of active IDH2 for proliferation and survival of glioblastoma cancer cells in hypoxia (48).

However, our primary goal was to evaluate the use of SLC25A1 as a therapeutic target to overcome radioresistance in chronically Hx lung cancer cells. It has been shown earlier that inhibition of SLC25A1 by BTA reduces the growth of xenograft tumors of breast, bladder or lung cancer cells without any evidence for additional normal tissue toxicity (39). But here we demonstrate for the first time that SLC25A1 has a role in lung cancer cell radiation resistance. For this we measured the effects of the SLC25A1-inhibitor BTA on radiation-induced eradication of clonogenic tumor cells in long-term colony formation assays (delayed plating) upon treatment in combination with IR. Our results revealed that treatment with BTA for 24 h was sufficient to significantly reduce the survival fraction of both, anoxia-tolerant NCI-H460 cells and the respective oxic NCI-H460 control cells. However, the radiosensitizing effect was more pronounced in the anoxia-tolerant NCI-H460 cells and could be further enhanced by treatment in acute severe hypoxia. Of note, we made similar observations when using a chemically distinct small molecule SLC25A1 inhibitor, CNASB, thereby corroborating the action of BTA at the level of SLC25A1. Moreover, the latter finding underlines the importance of SLC25A1-controlled metabolic and redox related alterations for adaptation to acute and chronic cycling hypoxia.

Mechanistically, we discovered that BTA exerts a dual effect on the cellular radiation response by targeting both, cell metabolism and DNA repair, making it a promising radiosensitizer. On the one hand, radiosensitizing effect of the SLC25A1-inhibitor BTA was associated with a reduction of the cellular antioxidant capacity: BTA treatment significantly reduced cellular total NADPH levels and increased NADP^+^/NADPH ratios in the anoxia-tolerant NCI-H460 cells. Moreover, BTA reduced GSH levels in both oxic and anoxia-tolerant NCI-H460 cancer cells, even in acute severe hypoxia. The reduction in these major determinants of cellular antioxidant capacity resulted in increased production of mitochondrial ROS and this effect on cellular redox-balance was even more pronounced when treatment had been performed in acute severe hypoxia. Our findings strongly suggest that SLC25A1 plays a role for maintenance of the antioxidant defense of lung cancer cells with tolerance to acute or chronic cycling severe hypoxia with potential impact on the sensitivity of the cells to ROS-induced damage. Our findings are in line with a report about a role of SLC25A1 for redox homeostasis in NCI-H460 cancer cells obtained by a stable knockout of SLC25A1 (36). Thus, our pharmacologic approach reproduces the findings obtained by the genetic approach, but shows in addition that such effects can also be obtained under conditions of acute severe hypoxia and in cells with tolerance to chronic cycling severe hypoxia. Importantly, the above-mentioned effects were neither due to direct toxic effects of BTA neither under Nx nor under Hx conditions as shown by the lack of a relevant increase in apoptosis (Figure S3 in Supplementary Material) or total cell death (not shown) upon acute treatment up to 72 h, nor to BTA-induced alterations in *SLC25A1* expression (Figure S1F in Supplementary Material).

Though SLC25A1 inhibition by BTA led to a significant induction of mitochondrial ROS compared to the respective untreated control cells, the percentage of cells stained positive for mitochondrial ROS was below 20% (Figure S2 in Supplementary Material). We therefore assume that inhibition of SLC25A1 might have effects in addition to the alterations in the redox balance that contribute to the cytotoxic effects BTA.

SLC25A1 is responsible for the bidirectional shuttling of citrate between the mitochondria and cytosol thereby supporting redox homeostasis by delivery of isocitrate to IDH2, which in turn regenerates NADPH. But SLC25A1 also impacts biosynthetic processes such as lipid biosynthesis (42) and other cellular processes. For example, it has been demonstrated that SLC25A1 and its function in citrate export to cytosol is central for cytokine-induced inflammatory signals and maintenance of NADPH redox state in macrophage activation (37, 49). Moreover, knockout of SLC25A1 has been associated with dysregulation of mitochondrial metabolism in cancer cells (44). Therefore, we further tested the effects of adaptation to chronic cycling severe hypoxia and BTA treatment on energy metabolism. Interestingly, anoxia-tolerant NCI-H460 cells displayed enhanced basal respiration and increased ability to respond to forced mitochondrial respiration (spare respiratory capacity) when compared to oxic NCI-H460 control cells. These findings demonstrate that the mitochondrial changes caused by adaptation to chronic cycling severe hypoxia are not restricted to components of the mitochondrial apoptosis signaling cascade as described earlier (22) but extend to alterations in mitochondrial oxidative metabolism. We also found that pharmacologic inhibition of SLC25A1 by BTA or CNASB significantly decreased mitochondrial respiration and ATP production. Interestingly, treatment with BTA or CNASB particularly lowered the spare respiratory capacity of anoxia-tolerant NCI-H460 cancer cells, pointing to a possible involvement of SLC25A1 in adaptation of oxidative metabolism to metabolic stress induced by chronic cycling severe hypoxia.

In addition, BTA treatment rendered NCI-H460 oxic and anoxia-tolerant cells more dependent on glycolysis-derived pyruvate and glutamine. It has been discussed that enhanced glucose uptake and a shift toward the pentose phosphate pathway (PPP) to generate more NADPH might be a strategy of cancer cells to counteract increased ROS (50). However, cancer cells use diverse strategies to increase their antioxidant capacity (cellular GSH) (31). For example, serine catabolism can participate in mitochondrial redox control under Hx conditions (51) whereas in our hands, altered glutamine usage contributed to the regeneration of glutathione and improved ROS defense of cancer cells with tolerance to cycling severe hypoxia (24). Excessive ROS-production—caused for example by oncogene-induced proliferation—causes oxidative damage of cellular macromolecules such as nuclear and mitochondrial DNA, membranes and proteins, thereby affecting major cell functions and cell survival (52–55). Moreover, there is an intimate link between adaptive changes in cell metabolism, generation of ROS, and the death threshold at the mitochondria by interconnected metabolic and redox sensitive pathways (56, 57). Therefore, the ability of the SLC25A1-inhibitor BTA to disrupt redox homeostasis makes it an attractive approach to enhance the cytotoxic effects of ROS-dependent treatments such as IR.

Finally, pharmacologic inhibition of SLC25A1 reduced the flexibility of oxic and anoxia-tolerant NCI-H460 cells to oxidize major metabolic fuels, which could in turn reduce their capability to meet altered nutrient availability after hypoxia-induced microenvironmental changes. Furthermore, BTA treatment decreased the capacity of NCI-H460 cells to oxidize FAs and also reduced FA uptake, particularly in anoxia-tolerant cells. Thereby our findings corroborate data obtained by genetic knockout of SLC25A1 in NCI-H460 cells revealing increased glycolysis and the usage of glutamine to compensate for the loss of FA synthesis as a consequence of reduced citrate transport (44). The same group further highlighted the complex functional role of SLC25A1 in glycolysis, redox homeostasis, and lipogenesis by quantitative metabolic flux analysis (44). Of note, dependency on the uptake of FAs has been recognized as a specific metabolic vulnerability of Hx cancer cells (58, 59).

Our metabolic investigations demonstrate that in addition to ensure the export of citrate from the mitochondria to the cytosol, e.g., for FA synthesis, SLC25A1 seems to be crucial for mitochondrial homeostasis and increased metabolic flexibility of the anoxia-tolerant NCI-H460 cells. Thereby our data confirm the assumption that SLC25A1 might be crucial for metabolic plasticity of cancer cells enabling adaptation and survival under adverse conditions such as nutrient starvation (glucose) or oxidative stress suggested by others (39, 47). But our data extend the stress conditions to acute and chronic cycling severe hypoxia. It appears that inhibition of SLC25A1 leads to a massive disturbance of mitochondrial metabolism and this might severely affect the ability of cancer cells to cope with the toxic effects of IR leading to radiosensitization and enhanced clonogenic cell death.

However, radiosensitivity is largely determined by the ability of the cells to repair radiation-induced DNA DSB. Analyzing the time-dependent formation and resolution of γH2AX –positive DNA repair foci as a mean of DNA-DSB we found that BTA treatment did not alter the initial amount of radiation-induced γH2AX -foci at 0.5–1 h after irradiation. However, BTA-mediated SLC25A1 inhibition slowed the kinetics of γH2AX-foci resolution, and this resulted in significantly higher levels of residual DNA damage in oxic and anoxia-tolerant NCI-H460 cells exposed to combined treatment when compared to irradiation alone at 2, 4, 6, and 24 h after irradiation.

Unexpectedly, we found that despite the obvious differences in radiosensitivity between anoxia-tolerant and oxic control NCI-H460 cells the kinetics in formation and resolution of γH2AX- foci indicative were rather similar in both cell lines. Therefore, it was not surprising that both cell lines responded similarly to combined treatment with BTA and IR with respect to the kinetics of the induction and repair of DNA DSB. From these data we conclude that other parameters of the DNA damage response such as their ability to cope with radiation-induced ROS might dictate the adaptation-induced differences in radiosensitivity and that the resulting differences in the extent of radiosensitization between oxic control and anoxia-tolerant cells might be due to drug-induced interference with redox homeostasis and disturbance of mitochondrial metabolism to meet cellular energy demands during the radiation response.

Nevertheless, these observations revealed that inhibition of SLC25A1 impacts DNA repair. Analyzing the underlying mechanisms we found that BTA-mediated inhibition of DSB repair correlated with an increase in D2-hydroxyglutarate levels induced by BTA treatment. In this context, BTA treatment led to elevated levels of D-2-HG in both NCI-H460 oxic and anoxia-tolerant cancer cells. Thereby, pharmacologic inhibition of SLC25A1 with BTA reproduces another metabolic effect of genetic inhibition of SLC25A1 where somatic loss of SLC25A1 induced a broad dysregulation of mitochondrial metabolism with accumulation of L- and D-enantiomers of 2-HG in NCI-H460 cells (44). Moreover, deletion of the SLC25A1 gene in humans causes the neurometabolic disorder D- and L-2-hydroxyglutaric aciduria, which is also characterized by increased accumulation of 2-HG (60, 61). These observations make it highly likely that elevated D-2-HG levels observed in response to BTA treatment are a direct and acute consequence of SLC25A1 inhibition.

Both enantiomers of 2-HG have been shown to promote malignant progression by their inhibitory action on α-ketoglutarate (αKG)-dependent dioxygenases; they are either synthesized as so-called “oncometabolite” as a result of gain-of-function mutations in IDH1/IDH2 (62, 63) or as pathologic metabolites in Hx cancer cells (64, 65).

Of note, accumulation of 2-HG has recently been linked to inhibition of DNA repair by inducing a homologous recombination repair defect thereby sensitizing cancer cells to poly (ADP-ribose) polymerase (PARP) inhibitors (46, 66). Furthermore, 2-HG was also shown to inhibit ALKBH DNA repair enzymes, leading to enhanced sensitivity to alkylating agents (67). Our new findings strongly suggest that BTA-induced accumulation of 2-HG might contribute to BTA-mediated radiosensitization and this finding could be exploited therapeutically in combination treatments.

Taken together, we identified a role of SLC25A1-regulated redox homeostasis in the tolerance of cancer cells to acute and chronic cycling hypoxia and increased radioresistance caused by adaptation to these stress conditions. In addition, our results clearly demonstrate that inhibition of SLC25A1 by the small molecule inhibitors BTA and CNASB is suited to increase radiosensitivity of NCI-H460 cancer cells exposed to acute or chronic cycling hypoxia. Mechanistically, treatment with the SLC25A1-inhibitor BTA disturbed cellular redox homeostasis by decreasing NADPH/GSH levels but also affected mitochondrial metabolism and cellular energy provision thereby reducing cancer cell survival (summarized in Figure 6F). Importantly, we demonstrate that BTA treatment impaired the repair of IR-induced DNA DSB and this was associated with elevation of D-2-HG levels. Since the method allowed us only to detect D-2-HG we cannot exclude that BTA treatment might also increase L-2-HG levels.

The pronounced effects of the SLC25A1-inhibitor BTA on cellular antioxidant capacity, cell metabolism and DNA repair make SLC25A1 inhibitors such as BTA interesting leads for the development of metabolic inhibitors of radioresistance. However, the development of small molecule inhibitors specifically targeting SLC25A1 at nanomolar drug concentrations is required for clinical translation of this approach.

Importantly, high expression of SLC25A1 in lung cancer patients was associated with a poor outcome, particularly after successful surgery (R0-resection), pointing to a potential clinical relevance of SLC25A1 particularly in terms of tumor recurrence. Moreover, exposure to IR, acute hypoxia, and chronic cycling severe hypoxia increased SLC25A1 expression in our *in vitro* models of anoxia-tolerant cancer cells. These findings suggest that SLC25A1 could be relevant as a potential biomarker of increased antioxidant capacity and metabolic flexibility and thus of a high potential for metabolic escape from radio (chemo)therapy.

We conclude that targeting the increased metabolic flexibility of cancer cells with tolerance to environmental stress, or of metabolic escape mechanisms ensuring DNA repair and cell survival under therapy, e.g., by pharmacologic inhibition of SLC25A1, represent attractive strategies to enhance vulnerability to genotoxic treatments and overcome microenvironment-mediated resistance to radio(chemo)therapy in advanced cancers.

## Materials and Methods

### Reagents and Cell Lines

If not stated otherwise, all chemicals were purchased from Sigma Aldrich (St. Louis, MO, USA). NCI-H460 lung adenocarcinoma cells, DU145 prostate cancer cells and T98G glioblastoma cells were obtained from ATCC (Bethesda, MD, USA) and were routinely tested for mycoplasma. NCI-H460, DU145 or T98G cells with tolerance to cycling severe hypoxia/reoxygenation stress were generated by exposure to 16 cycles (T98G) or 25 cycles (NCI-H460, DU145) of severe hypoxia (48 h, <0.1% O_2_) and reoxygenation (120-h air plus 5% CO_2_ referred as 20% O_2_) as described earlier (24). These cells hypoxia/reoxygenation-tolerant cells will be termed “anoxia-tolerant cells” throughout the manuscript. Control cells were cultured in parallel under standard ambient O_2_ conditions (20% O_2_ plus 5% CO_2_; the control cells will be termed “oxic cells” throughout the manuscript) (24, 59). Upon selection, cancer cells were routinely grown in RPMI 1640 medium supplemented with 10% (v/v) fetal calf serum (Gibco/Life Technologies, Carlsbad, CA, USA) and maintained in a humidified incubator at 37°C and 5% CO_2_ (referred to as “normoxia” or “Nx conditions,”). For severely Hx conditions cells were grown in a humidified hypoxia work station (*In vivo* 400, Ruskinn Technology Ltd., Bridgend, Great Britain) at 37°C, 0.2% O_2_, and 5% CO_2_ (referred to as “hypoxia” or “Hx conditions,”).

### Patient Survival Data

Patient array data were obtained from and analyzed by Kaplan–Meier plotter tool (kmplot.com) as described elsewhere (40, 41). The cohort was split by median of SLC25A1 expression (“High” and “Low,” respectively). Analysis was performed in the cohort once without additional restrictions and once including only patients which had successful surgery (only surgical margins negative), as described in detail elsewhere (40). For further details of used settings please refer to Tables S1 and S2 in Supplementary Material.

### qRT-PCR Analysis

cDNA was synthesized from 1 µg of total RNA using QuantiTect Reverse Transcription Kit (Qiagen, Hilden, Germany) according to the manufacturer’s protocol. Specific primers were synthesized based on available sequences for each listened gene. Primer sets for qRT-PCR were designed with the Blast web tool (U.S. National Center for Biotechnology Information, Bethesda, MD, USA). To exclude cross-reaction of primers with the genes the sequence of interest was compared as well with the Blast database. PCR products were 150–200 bp in size. We used published β2-microglobulin (B2M) primer sequences as housekeeping gene (68). qRT-PCR and cycling conditions were performed using specific oligonucleotide primers (*B2M* forward: TGCTGTCTCCATGTTTGATGTATCT; reverse: TCTCTGCTCCCCACCTCTAAGT; *SLC25A1* forward: CAACGGGGTGAGGGCAT; reverse: CTCGGTGGGGAAGGTGATG; *IDH1* forward: CTCTGTGGCCCAAGGGTATG; reverse: GGATTGGTGGACGTCTCCTG; IDH2 forward: CCTGCTCGTTCGCTCTCC; reverse: GCTTCGCCACCTTGATCCT) and using qPCR kit for SYBR^®^ Green I, 6-Carboxyl-X-Rhodamine (ROX) (Eurogentec, Cologne, Germany) according to the manufacturers protocol. Reactions were carried out on an ABI Prism 7900HT using MicroAmp Optical 384 well Reaction plate (Applied Biosystems by Life Technologies, Bleijswijk, Netherlands) and BIO-RAD PCR Sealers Microseal “B” Film Adhesive seal (optically clear; BIORAD, Munich, Germany). Melting curves were obtained after each PCR run and showed single PCR products. cDNAs were run in triplicate, without reverse transcriptase and no-template controls were run in duplicates. Expression levels for the genes of interest and for the housekeeping gene *B2M* were measured in three independent PCR runs. Expression ratios were calculated using the geometric mean expression of the housekeeping gene *B2M* to normalize the expression data for the genes of interest according to the 2^-ΔΔCt^—method as described by others (69).

### Western Blot Analysis

Anti-rabbit SLC25A1 Polyclonal antibody (Thermo Fisher, Rockford, IL, USA) and anti-mouse β-actin from Sigma Aldrich (St. Louis, MO, USA) were used for Western blot analysis. After harvesting, cells were lysed in 75 µL of RIPA buffer containing 0,5% Sodiumdesoxycholate, 1% NP-40 (Nonidet), 0,1% Sodiumdodecylsulfate (SDS), 50-mM Tris-HCl, 150-mM NaCl, 5-µg/mL aprotinin, 5-µg/mL leupeptin, and 3-µg/mL pepstatin. Protein was separated by 12% sodium dodecyl sulfate-polyacrylamide gel electrophoresis (SDS-PAGE) under reducing conditions and transferred onto polyvinylidene fluoride (PVDF) membranes (Roth, Karlsruhe, Germany). Blots were blocked in RotiBlock (Roth, Karlsruhe, Germany) for 1 h at room temperature. The membranes were incubated overnight at 4°C with the respective primary antibodies. The secondary antibody was incubated for 1 h at room temperature. Detection of antibody binding was performed by enhanced chemiluminescence (ECL Western Blotting Analysis System; GE Healthcare/Amersham Biosciences, Freiburg, Germany). Equal loading was verified by antibodies against β-actin. Densitometry analysis was performed using ImageJ 2.00 (National Institutes of Health, Bethesda, MD, USA).

### Determination of Redox Homeostasis

NADPH levels were measured using NADPH Glo Assay (Promega, Madison, WI, USA) according to manufacturer’s protocol. Briefly, adherent cells in 96-well plates were lysed and heated under acidic and basic conditions to measure NADP^+^ and NAPH individually using a luciferase-coupled enzymatic reaction. In parallel, technical replicates were fixed with 4% paraformaldehyde and stained with 10-µg/mL solution of fluorescent dye Hoechst 33342 (Thermo Scientific, Waltham, MA, USA) for normalization to DNA content. Luminescence and Flourescence were measured in triplicates using a BioTek Synergy Microplate reader (BioTek, Winooski, NH, USA).

Levels of reduced Glutathione (GSH) were determined by using Monochlorobimane (MCB) which was described to be specific for GSH metabolized by Glutathione-S-Transferase, leading to a fluorescent adduct (70). The published protocol for measurement of GSH in a Microplate Reader (71) was adapted for using 10-µM MCB with 15 min of preincubation. To rule out alterations in the speed of dye metabolism instead of alterations in absolute cellular GSH levels, kinetic measurements of fluorescence were performed for 15 min at 37°C. Each assay contained cells treated with H_2_O_2_ to deplete GSH as a negative control.

To quantify mitochondrial ROS production, cells were stained for 30 min at 37°C with 5 µM of MitoSox (Molecular Probes/Invitrogen, Carlsbad, CA, USA). To quantify cellular ROS production, cells were stained for 30 min at 37°C with 5 µM of Dihydroethidium (DHE) (Molecular Probes/Invitrogen, Carlsbad, CA, USA). MitoSox- or DHE-positive cells were detected by flow cytometry (BD Accuri C6, Becton Dickinson, Heidelberg, Germany; FL-2). Fractions of gated positive cells (at least 10,000) with higher fluorescence were evaluated. Fold changes were quantified to the corresponding controls.

### Extracellular Flux Analysis

NCI-H460 cells were plated at 7.500 cells/well in XF96 microplates (Seahorse Bioscience, Billerica, MA, USA) in RPMI Medium with 10% FCS according to manufacturer’s recommendations 48 h prior to the assay. Treatment with 5 mM BTA was performed for 24 h. One hour prior to the assay, medium was exchanged to XF base medium (Seahorse Bioscience, Billerica, MA, USA) with 1-mM Pyruvate, 2-mM Glutamine and 10-mM Glucose and incubated at 37°C without CO_2_. During assays, OCR was measured using a Seahorse XFe 96 analyzer_._ Mito Stress Test Kit containing 1-µM Oligomycin, 2-µM Carbonyl cyanide-*4*-(trifluoromethoxy)phenylhydrazone (FCCP), 0.5-µM Rotenone and 0.5-µM Antimycin A was perfomed according to manufacturer’s protocol. For individual normalization to DNA content, fluorescence was measured after cells were fixed with 4% PFA and stained with 10-µg/mL Hoechst 33342 solution after each assay. Fuel Flex Test Kit containing 3-µM BPTES, an inhibitor of glutaminase (GLS1), 4-µM etomoxir, an inhibitor of carnitine palmitoyl-transferase 1 A (CPT1A) and 2-µM UK5099, an inhibitor of the mitochondrial pyruvate carrier (MPC), was also performed according to manufacturer’s protocol. Data were analyzed using Wave 2.4 software (Seahorse Bioscience, Billerica, MA, USA).

### Determination of Fatty-Acid Uptake

The uptake of FA was quantified by using fluorescent FA analog C1-BODIPY^®^ 500/510 C12. In brief, fluorescent FA (5 µM) were added 24 h after treatment with 5-mM BTA to serum-free media. We quenched the fluorescence of FA in media by adding trypan blue (0.33 mM) to the media. The uptake of fluorescent FA was measured after 1 h, at 37°C spectrophotometrically at 485/528 nm (59). For individual normalization to DNA content, Hoechst 33342 fluorescence was measured after the assay. Cells were fixed with 4% PFA and stained with 10-µg/mL Hoechst 33342 solution.

### Quantification of D-2-Hydroxyglutarate

Levels of D-2-HG were measured using colorimetric D-2-Hydroxyglutarate Assay Kit (BioVision, Milpitas, CA, USA) according to manufacturer’s protocol. At a glance, 10^7^ cells were lysed, spun down and supernatant was transferred into a 96-well plate to quantify the enzymatic conversion of D-2-Hydroxyglutarate to α-Ketoglutarate leading to colored product which is detected photometrically at 450 nm using a BioTek Synergy Microplate reader (BioTek, Winooski, NH, USA).

### Irradiation and Treatment

1,2,3-benzene-tricarboxylic acid and CNASB were purchased from Sigma-Aldrich (St. Louis, MO, USA). Stock solutions of 200-mM BTA were made with phosphate-buffered aqua bidest and 14-mM CNASB was solved in DMSO. Irradiation was performed at room temperature with an X-ray machine (Precision X-Ray Inc., North Branford, CT, USA) operated at 320 kV, 12.5 mA with a 1.65-mm Al filter, at a distance of 50 cm and a dose rate of 3.71 Gy/min. For an irradiation dose of 2 Gy, the cells were irradiated approximately 32 s, for 5 Gy approximately 81 s. Cells were returned to the incubator immediately after exposure to IR. For irradiation under Hx conditions, Hx cell dishes were kept in BD GasPak EZ Pouch System (Becton Dickinson, Heidelberg, Germany). In terms of combined treatments, inhibitors were added 2 h prior to irradiation.

### Immunofluorescence: γH2AX Assays

Cells were seeded on glass coverslips placed in 12-well plates and irradiated 24 h later with 5 Gy. Next, cells were fixed and permeabilized in 3% PFA/0.2% Triton-X100 for 15 min and incubated in blocking solution including 2% goat serum at room temperature for 1 h. γH2AX foci were stained for 1 h at room temperature (RT) with Alexa Fluor^®^ 647 mouse anti-H2A.X (pS139) (BD Biosciences, San Jose, CA, USA) diluted 1:50 in blocking solution. DNA was stained with Hoechst33342 (3 µM in PBS) for 30 min at RT. Coverslips were mounted onto glass slides with DAKO mounting medium (Dako NA Inc., Carpinteria, CA, USA). Slides were analyzed with a Zeiss Axiovert 200 fluorescence microscope with ApoTome and ZEN imaging software (Carl Zeiss, Goettingen, Germany). γH2AX foci in at least 50 cells per slide were counted with the Focinator software developed in our laboratory (72, 73).

### Colony Formation Assays

Clonogenic cell survival in response to the respective treatments was determined comparing the clonogenic survival of cells cultured under Nx and severely Hx conditions. For treatment in normoxia, exponentially growing cells were seeded in tissue culture flasks, incubated under standard culturing conditions (20% O_2_, 5% CO_2_, 37°C) and irradiated 24 h later (0 to 5 Gy) without or with prior BTA treatment (5 mM). BTA treatment was performed 2 h prior to irradiation. For treatment in hypoxia, tissue culture flasks of exponentially growing cells were exposed to severe hypoxia (0.2% O_2_) 2 h prior to BTA treatment and 4 h prior to irradiation, respectively. After completion of the treatments, cells were incubated for 24 h under Nx or Hx conditions, respectively, then washed, collected (0.05% Trypsin, 0.01% EDTA), and plated to 6-well plates at densities of 200–3,200 cells per well (delayed plating). The cell viability was checked before plating the cells by using CASY COUNT (Omni Life Science, Bremen, Germany) and only the number of viable cells was plated. Plates were subsequently incubated for 9 days under standard Nx conditions before quantification of colony formation. For this, cells were fixed in 3.7% formaldehyde and 70% ethanol, stained with 0.05% Coomassie blue, and colonies of at least 50 cells were counted by GelCount (Oxford Optronix, Oxfordshire, Great Britain). The plating efficiency and surviving fraction (SF) to corresponding Nx and Hx controls were calculated as described elsewhere (74).

### Toxicity Testing

For quantification of apoptotic DNA-fragmentation (sub-G1 population), cells were incubated for 30 min at room temperature with a staining solution containing 50-µg/mL PI in a hypotonic citrate buffer 0.1% (w/v) sodium citrate and 0.05% (v/v) Triton X-100 and subsequently analyzed by flow cytometry (BD Accuri C6, Becton Dickinson, Heidelberg, Germany; FL-2) (75).

For determination of cell proliferation and viability, cells were washed with PBS (1x), fixed with Glutaraldehyde (0.1% in PBS), and stained with crystal violet (0.1% in PBS). The dye was released by TritonX-100 (0.2% in PBS) and measured spectrophotometrically at 540 nm as described elsewhere (76).

### Statistics

Data represent mean values of at least three independent experiments ± SEM except for Figures 5A,B which show data from *n* = 12–18 wells from two independent experiments. Data analysis was performed either by two-way ANOVA test using parametric methods and employing Tukey multiple comparison post-test where appropriate or by unpaired Student’s *t*-test using Prism6 software (Graph Pad Inc., La Jolla, CA, USA). The values of *P* ≤ 0.05 were considered significant.

## Author Contributions

JM, JH, and VJ designed and conceptualized the research. JH, CH, and JM performed experiments, analyzed, validated, and visualized the results. JM, JH, and VJ wrote the original manuscript draft. JM and VJ supervised the work. VJ acquired the funding. All authors critically revised, edited, and approved the final version of the manuscript.

## Conflict of Interest Statement

The authors declare that the research was conducted in the absence of any commercial or financial relationships that could be construed as a potential conflict of interest.
