# Supplementary material for: The Mitochondrial Citrate Carrier (SLC25A1) Sustains Redox Homeostasis and Mitochondrial Metabolism Supporting Radioresistance of Cancer Cells With Tolerance to Cycling Severe Hypoxia
Source: Front Oncol. 2018 May 25;8:170. doi: 10.3389/fonc.2018.00170 (PMC5980958; doi:10.3389/fonc.2018.00170)
Supplement: Supplementary file 1 [file Data_Sheet_1.docx]

Supplementary Material

The mitochondrial citrate carrier (SLC25A1) sustains redox homeostasis and mitochondrial metabolism supporting radioresistance of cancer cells with tolerance to cycling severe hypoxia.

Julian Hlouschek, Christine Hansel, Verena Jendrossek, Johann Matschke

*** Correspondence:**

Dr. Johann Matschke, Institute of Cell Biology (Cancer Research), University of Duisburg-Essen, University Hospital Essen, Essen, Germany
[johann.matschke@uk-essen.de](mailto:johann.matschke@uk-essen.de)

## Supplementary Figures


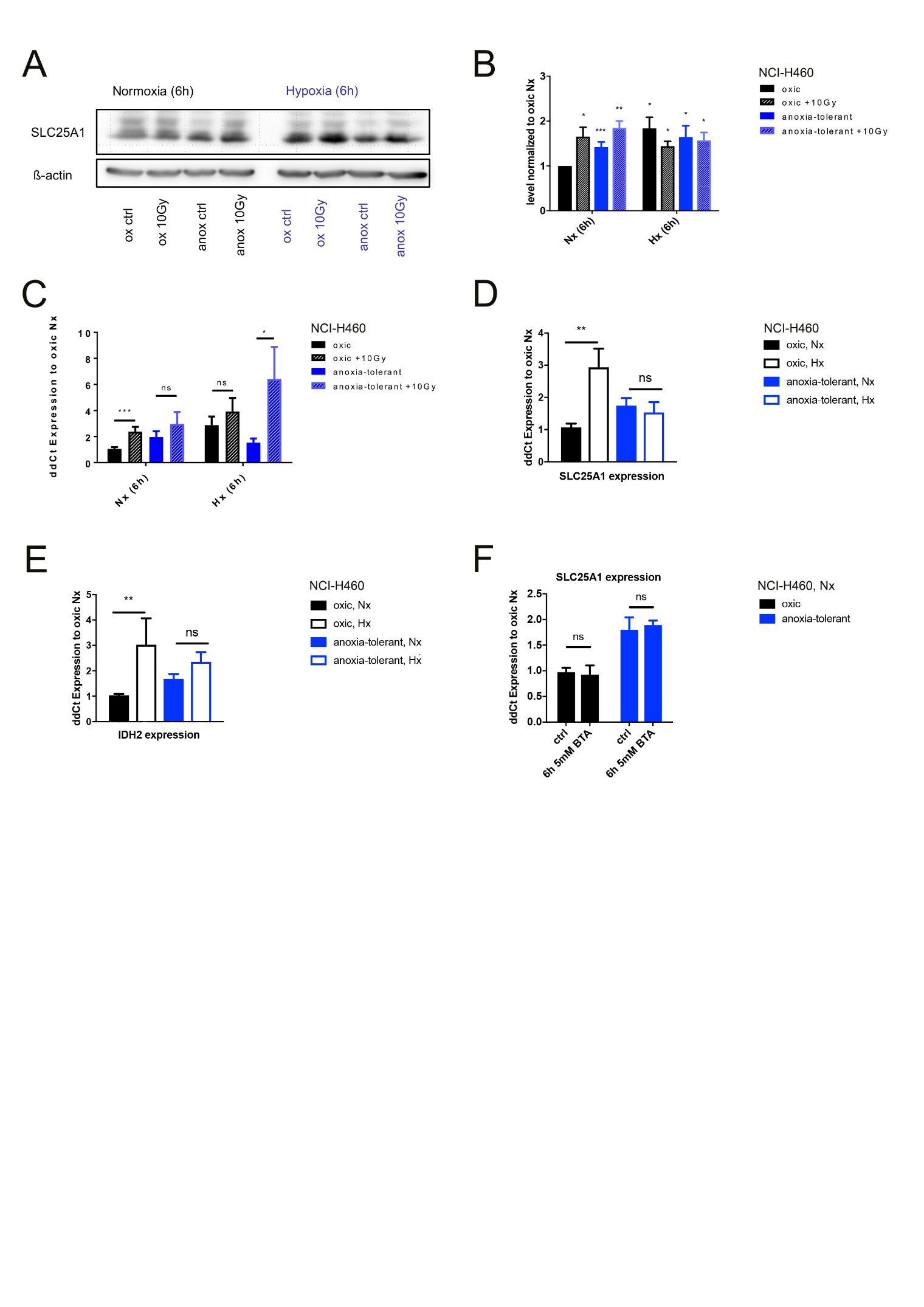


**Supplementary Figure 1.** Anoxia-tolerant NCI-H460 cells (anox) and oxic NCI-H460 control cells (ox) were cultured in normoxia (20% O_2_) or severe hypoxia (0.2% O_2_), irradiated and collected 6h after irradiation for the generation of cell lysates. A) Data show representative Western blots detecting the amount of SLC25A1 protein in lysates in cells cultured upon above mentioned conditions. B) Quantification of relative SLC25A1 protein expression detected by Western blot analyses. Comparisons to oxic Nx control are indicated above each bar. C) qRT-PCR validation of *SLC25A1* expression levels 6h after IR with 10Gy under normoxic (Nx, 20% O_2_) and hypoxic (Hx, 0.2% O_2_) conditions. D) Comparison of SLC25A1 expression upon 6h of exposure to hypoxia (Hx) compared to oxic Nx control determined by qRT-PCR. E) Comparison of IDH2 expression upon 24h of exposure to hypoxia (Hx) compared to oxic Nx control determined by qRT-PCR. F) Comparison of SLC25A1 expression upon 6h of treatment with 5mM BTA under normoxic (Nx) conditions compared to oxic Nx control determined by qRT-PCR. Mean values ±SEM are shown, n=3 (ns p>0.05, * *p*≤0.05, ** p < 0.01, *** *p*≤0.001; t-test).


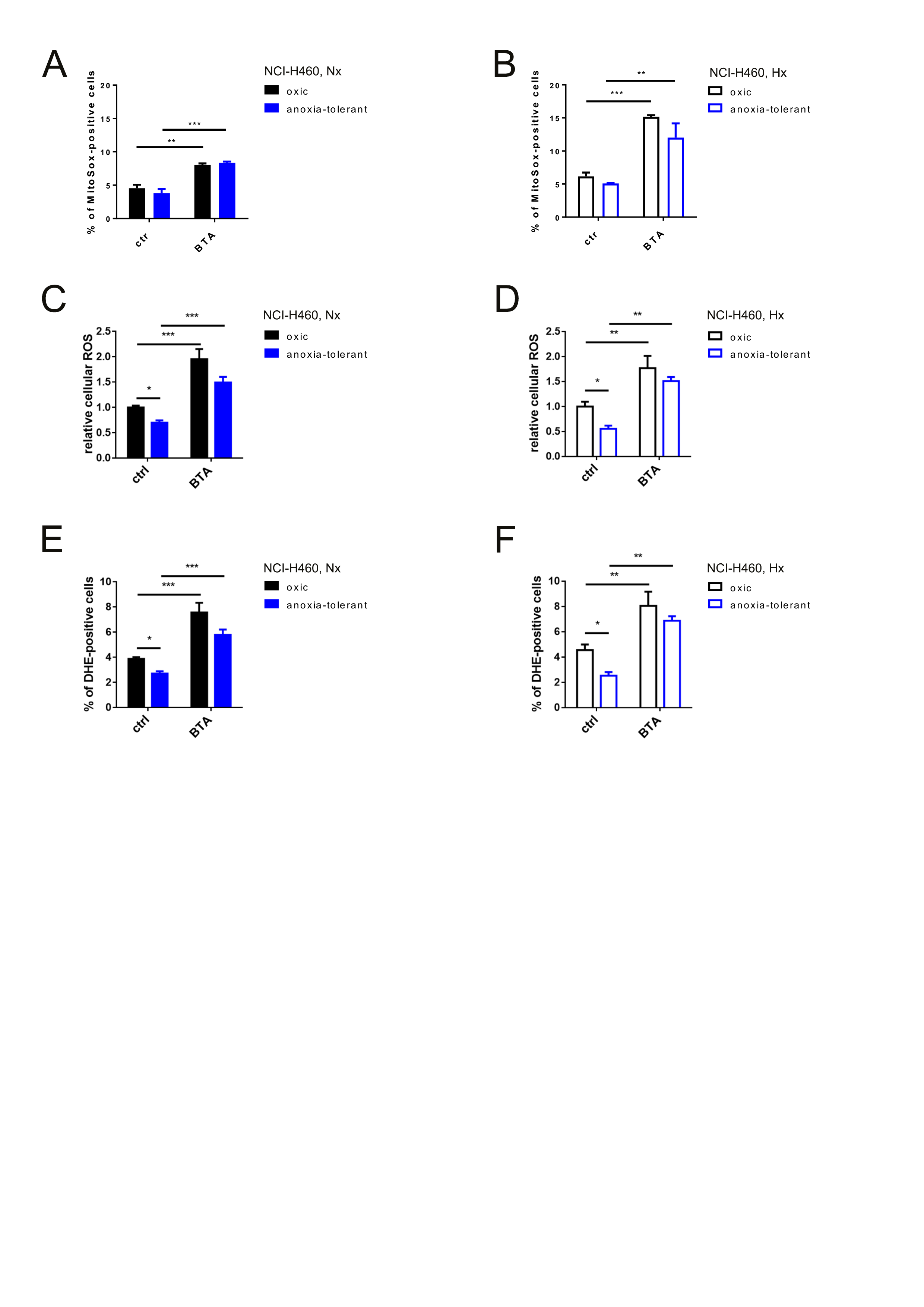


**Supplementary Figure 2.** NCI-H460 oxic and anoxia-tolerant cells were treated with 5mM BTA under normoxic (20% O_2_) or hypoxic conditions (0.2% O_2_), and mitochondrial superoxide reactive oxygen species (ROS) or cellular ROS were determined by flow cytometry upon cell staining with MitoSox or DHE. A) Mitochondrial ROS (Fraction (%) of gated MitoSox-positive cells) induced by 6h of BTA treatment in normoxia (Nx) B) Mitochondrial ROS (Fraction (%) of gated MitoSox-positive cells) induced by 6h of BTA treatment in hypoxia (Hx). C) Cellular ROS (relative to oxic control) induced by 24h of BTA treatment in normoxia (Nx) D) Cellular ROS (relative to oxic control) induced by 24h of BTA treatment in hypoxia (Hx). E) Cellular ROS (Fraction (%) of gated DHE-positive cells) induced by 24h of BTA treatment in normoxia (Nx) F) Cellular ROS (Fraction (%) of DHE-gated positive cells) induced by 24h of BTA treatment in hypoxia (Hx). Fraction (%) of gated positive cells (at least 10.000) are indicated. Mean values ±SEM are shown, n=3 (* *p*≤0.05, ** p < 0.01, *** *p*≤0.001; two-way ANOVA with Tukey post-test).


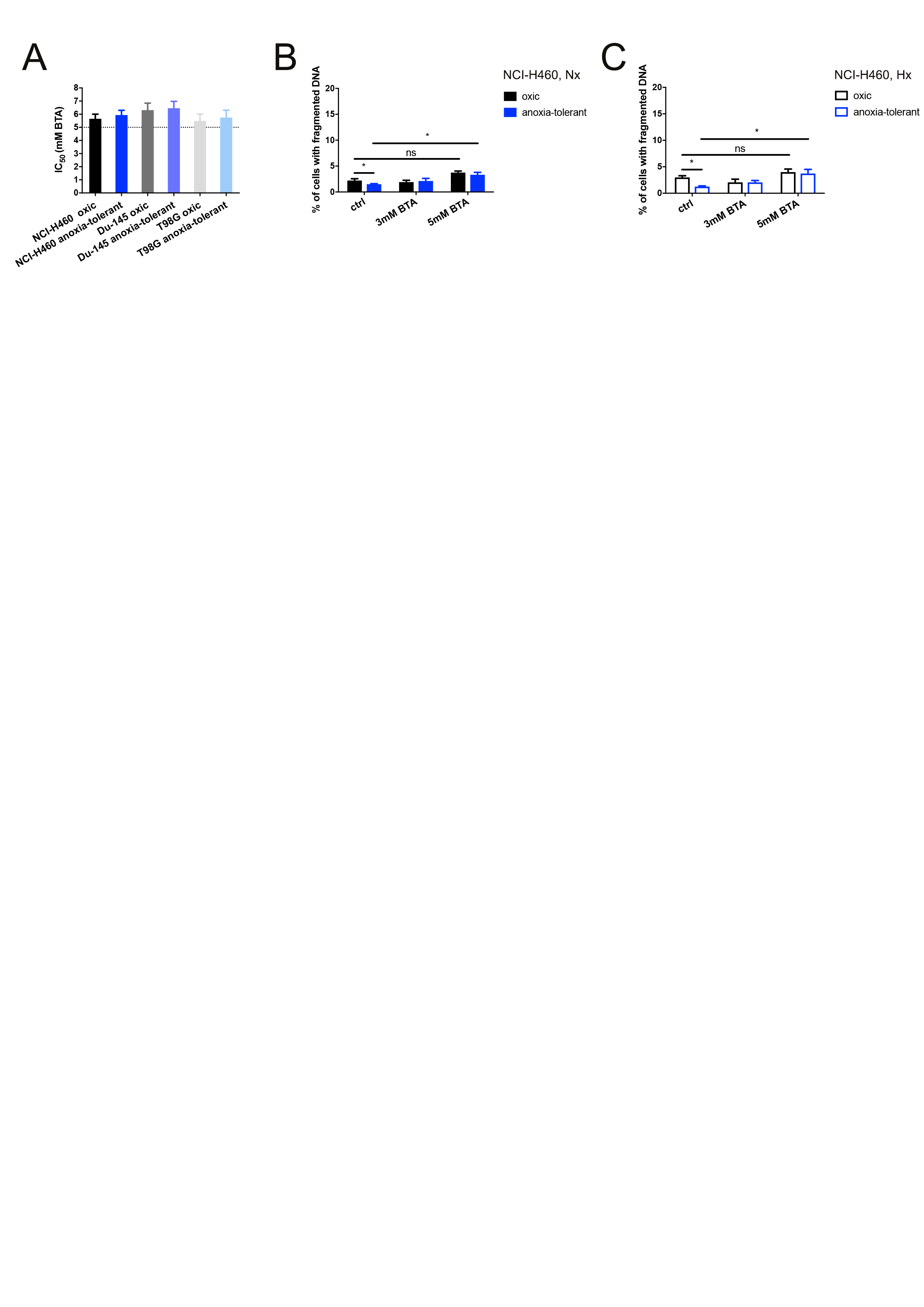


**Supplementary Figure 3.** Oxic and anoxia-tolerant cells were treated for 72h with different concentrations of BTA under normoxic (20% O_2_) or hypoxic conditions (0.2% O_2_). Apoptotic cells with fragmented DNA were determined by flow cytometry and proliferation or cell viability were determinded by crystal violet staining. A) Inhibitory concentrations of 50% proliferation reduction (IC_50_) were determined by crystal violet staining under normoxia. B) NCI-H460 oxic and anoxia-tolerant cells with fragmented DNA upon BTA treatment in normoxia (Nx) were determined by flow cytometry. C) NCI-H460 oxic and anoxia-tolerant cells with fragmented DNA upon BTA treatment in hypoxia (Hx) were determined by flow cytometry. Fraction (%) of gated positive cells (at least 10.000) are indicated. Mean values ±SEM are shown, n=3 (* *p*≤0.05, ** p < 0.01, *** *p*≤0.001; two-way ANOVA with Tukey post-test).


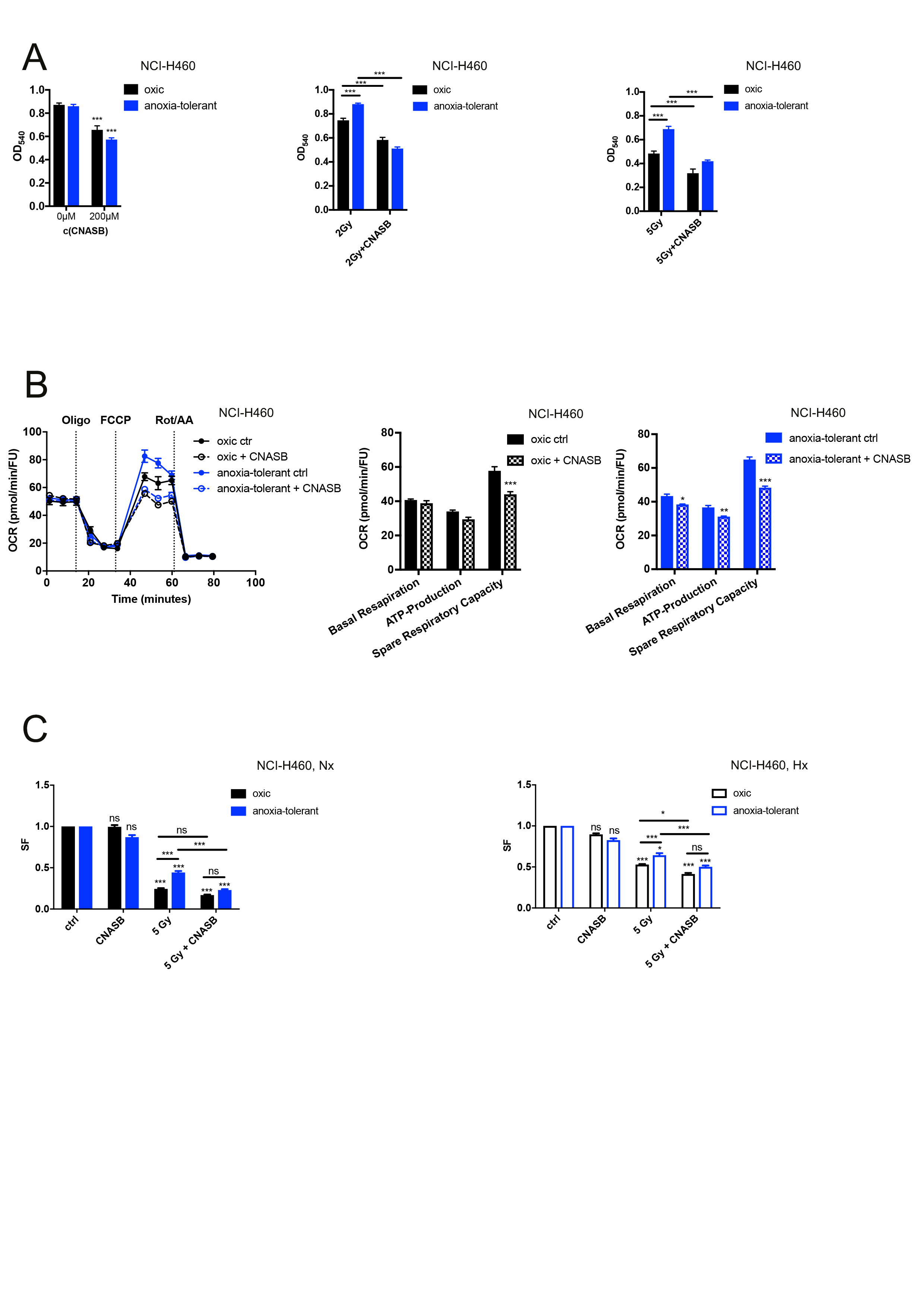


**Supplementary Figure 4.** NCI-H460 oxic and anoxia-tolerant cells were treated with 200µM of 4-Chloro-3-[[(3-nitrophenyl)amino]sulfonyl]-benzoic acid (CNASB), Inhibitor of SLC25A1 under normoxic (Nx, 20% O_2_) or hypoxic conditions (Hx, 0.2% O_2)_). In case of combined treatment with ionizing radiation (IR), 2h of pretreatment CNASB were performed. A) Influence of CNASB alone or in combination with IR on cell proliferation after 72h were determined by crystal violet staining and measurement of optic density at 540nm (OD_540_) after cell-lysis. B) Oxygen consumption rate (OCR) normalized to Hoechst 33342 fluorescence units (FU) 24h after treatment with CNASB was measured using Seahorse XFe 96 analyzer and Mito Stress Test Kit. Real-time Injection of Oligomycin (Oligo, 1µM), Carbonyl cyanide-*4*-(trifluoromethoxy)phenylhydrazone (FCCP, 2µM), Rotenone (Rot, 0.5µM) and Antimycin A (AA, 0.5µM). Calculated parameters of the assay are indicated in bar graphs. C) Anoxia-tolerant NCI-H460 cells and the oxic NCI-H460 control cells were pretreated with 200µM BTA (2h prior to IR) under normoxic condition (Nx, 20% O_2_) or upon 2h pre-incubation in severe hypoxia (Hx, 0.2% O_2_). 24h after treatment cells were collected, plated at different cell numbers (200-3200) in full medium without the inhibitor and grown under Nx for 9 days. Bars depict quantification of colony formation upon treatment with CNASB alone or in combination with IR in normoxia (Nx) and severe hypoxia (Hx). Colonies were scanned and counted using GelCount. Survival fractions (SF) were calculated to the plating efficiency of unirradiated cells under normoxic or hypoxic conditions. Mean values ±SEM are shown, n=3 (* *p*≤0.05, ** p < 0.01, *** *p*≤0.001; two-way ANOVA with Tukey post-test).

## Supplementary Tables

**Supplementary Table 1.** Table showing the parameters for the *in silico* analysis of overall survival (OS) of cohort of patients with SLC25A1 up-regulation performed by KMPlotter.

| Affy ID | 210010_s_at (SLC25A1; CTP; SLC20A3) |
| --- | --- |
| Backward reference | CaArray |
|  | GSE14814 |
|  | GSE19188 |
|  | GSE29013 |
|  | GSE30219 |
|  | GSE31210 |
|  | GSE3141 |
|  | GSE31908 |
|  | GSE37745 |
|  | GSE4573 |
|  | GSE50081 |
|  | TCGA |
| Survival | OS |
| Split patients by | median |
| Follow up threshold all | all |
| Censore at threshold | checked |
| Compute median over entire database | false |
| Cutoff value used in analysis | 634 |
| Expression range of the probe | 21 - 4503 |
| Probe set option | user selected probe set |
| Invert HR values below 1 | not checked |
| Restrictions |  |
| Histology | all |
| Grade | all |
| Stage | all |
| AJCC stage T | all |
| AJCC stage N | all |
| AJCC stage M | all |
| Gender | all |
| Smoking history | all |
| Surgery success | all |
| Chemotherapy | all |
| Radiotherapy | all |
| Dataset | all |
| Use earlier release of the database | 2015 version (n=2437) |
| Array quality control | exclude biased arrays |

**Supplementary Table 2.** Table showing the the parameters for the *in silico* analysis of overall survival (OS) of cohort of patients with SLC25A1 up-regulation and successful surgery with tumor-free margins (R0-resection) performed by KMPlotter.

| Affy ID | 210010_s_at (SLC25A1; CTP; SLC20A3) |
| --- | --- |
| Backward reference | CaArray |
|  | GSE31210 |
|  | TCGA |
| Survival | OS |
| Split patients by | median |
| Follow up threshold | all |
| Censore at threshold | checked |
| Compute median over entire database | false |
| Cutoff value used in analysis | 579 |
| Expression range of the probe | 93 - 4503 |
| Probe set option | user selected probe set |
| Invert HR values below 1 | not checked |
| Restrictions |  |
| Histology | all |
| Grade | all |
| Stage | all |
| AJCC stage T | all |
| AJCC stage N | all |
| AJCC stage M | all |
| Gender | all |
| Smoking history | all |
| Surgery success | only surgical margins negative |
| Chemotherapy | all |
| Radiotherapy | all |
| Dataset | all |
| Use earlier release of the database | 2015 version (n=2437) |
| Array quality control | exclude biased arrays |
